# Direct visualization of degradation microcompartments at the ER membrane

**DOI:** 10.1073/pnas.1905641117

**Published:** 2019-12-27

**Authors:** Sahradha Albert, Wojciech Wietrzynski, Chia-Wei Lee, Miroslava Schaffer, Florian Beck, Jan M. Schuller, Patrice A. Salomé, Jürgen M. Plitzko, Wolfgang Baumeister, Benjamin D. Engel

**Affiliations:** ^a^Department of Molecular Structural Biology, Max Planck Institute of Biochemistry, 82152 Martinsried, Germany;; ^b^Department of Structural Cell Biology, Max Planck Institute of Biochemistry, 82152 Martinsried, Germany;; ^c^Department of Chemistry and Biochemistry, University of California, Los Angeles, CA 90095

**Keywords:** proteasome, cdc48, ERAD, phase separation, cryo-electron tomography

## Abstract

To promote the biochemical reactions of life, cells can compartmentalize molecular interaction partners together within separated non–membrane-bound regions. It is unknown whether this strategy is used to facilitate protein degradation at specific locations within the cell. Leveraging in situ cryo-electron tomography to image the native molecular landscape of the unicellular alga *Chlamydomonas reinhardtii*, we discovered that the cytosolic protein degradation machinery is concentrated within ∼200-nm foci that contact specialized patches of endoplasmic reticulum (ER) membrane away from the ER–Golgi interface. These non–membrane-bound microcompartments exclude ribosomes and consist of a core of densely clustered 26S proteasomes surrounded by a loose cloud of Cdc48. Active proteasomes in the microcompartments directly engage with putative substrate at the ER membrane, a function canonically assigned to Cdc48. Live-cell fluorescence microscopy revealed that the proteasome clusters are dynamic, with frequent assembly and fusion events. We propose that the microcompartments perform ER-associated degradation, colocalizing the degradation machinery at specific ER hot spots to enable efficient protein quality control.

## Significance

Endoplasmic reticulum-associated degradation (ERAD) is an essential process that removes misfolded proteins from the ER, preventing cellular dysfunction and disease. While most of the key components of ERAD are known, their specific localization remains a mystery. This study uses in situ cryo-electron tomography to directly visualize the ERAD machinery within the native cellular environment. Proteasomes and Cdc48, the complexes that extract and degrade ER proteins, cluster together in non–membrane-bound cytosolic microcompartments that contact ribosome-free patches on the ER membrane. This discrete molecular organization may facilitate efficient ERAD. Structural analysis reveals that proteasomes directly engage ER-localized substrates, providing evidence for a noncanonical “direct ERAD” pathway. In addition, live-cell fluorescence microscopy suggests that these ER-associated proteasome clusters form by liquid–liquid phase separation.

The ubiquitin–proteasome system degrades both unwanted and misfolded proteins, playing a vital role in maintaining proteostasis and regulating numerous processes throughout the cell. Within the nucleus, proteasomes are often concentrated in “degradation centers,” regions of high degradation capacity. In mammalian cells, nuclear proteasomes can accumulate in non–membrane-bound compartments called PML bodies (1). In yeast, *Drosophila* S2 cells, and the green alga *Chlamydomonas reinhardtii*, nuclear proteasomes accumulate at the periphery of the nucleus (2–4), which in *Chlamydomonas* was shown to be mediated by tethering proteasomes to the nuclear pore complex (NPC) (5). However, it is unknown whether cytosolic proteasomes also accumulate to form degradation centers. One hint that cytosolic proteasomes might have specific cellular localization is their requirement for the elimination of misfolded proteins from the endoplasmic reticulum (ER).

Approximately one-third of the proteins in a eukaryotic cell are synthesized by ER-bound ribosomes and inserted into either the ER membrane or lumen (6, 7), where they are folded and then trafficked through the secretory system to a variety of intracellular and extracellular destinations. The accumulation of misfolded proteins in the ER is toxic to the cell and underlies numerous human diseases (8). Proteins that fail to be refolded by ER-localized chaperones (9) are eliminated by an evolutionarily conserved quality control pathway called ER-associated degradation (ERAD) (10–12). Misfolded proteins are retrotranslocated to the cytosol and polyubiquitinated by the channel-forming E3 ligase Hrd1 (13, 14). The type II AAA+ segregase, Cdc48, is recruited by Ubx2 to the ER membrane (15), where it is believed to play a key role in pulling these polyubiquitinated substrates away from the Hrd1 channels so that they can be degraded by cytosolic 26S proteasomes (16–18). While the major players in ERAD have been identified, little is known about how they are spatially organized within the cell. Some ERAD proteins appear to concentrate in a subcompartment of the mammalian ER (19), and a fraction of cytosolic proteasomes may associate with the ER membrane (3, 20). However, it remains a mystery whether ERAD is performed uniformly throughout the ER network, or whether it is coordinated in specialized regions.

To explore the cellular organization of the cytosolic ubiquitin–proteasome system, we combined focused ion beam (FIB) milling (21–23) with cryo-electron tomography (cryo-ET) to directly visualize macromolecules in situ, within the native cellular environment (24). By imaging the model green alga *Chlamydomonas reinhardtii*, which has conserved ERAD components (*SI Appendix*, Figs. S1 and S2 and Table S1), modern genetic tools (25), excellent cryo-EM contrast, and textbook organelle architecture (26–28), we found that proteasomes and Cdc48 cluster together in non–membrane-bound microcompartments that contact the ER membrane.

## Results

**Fig. 1.**
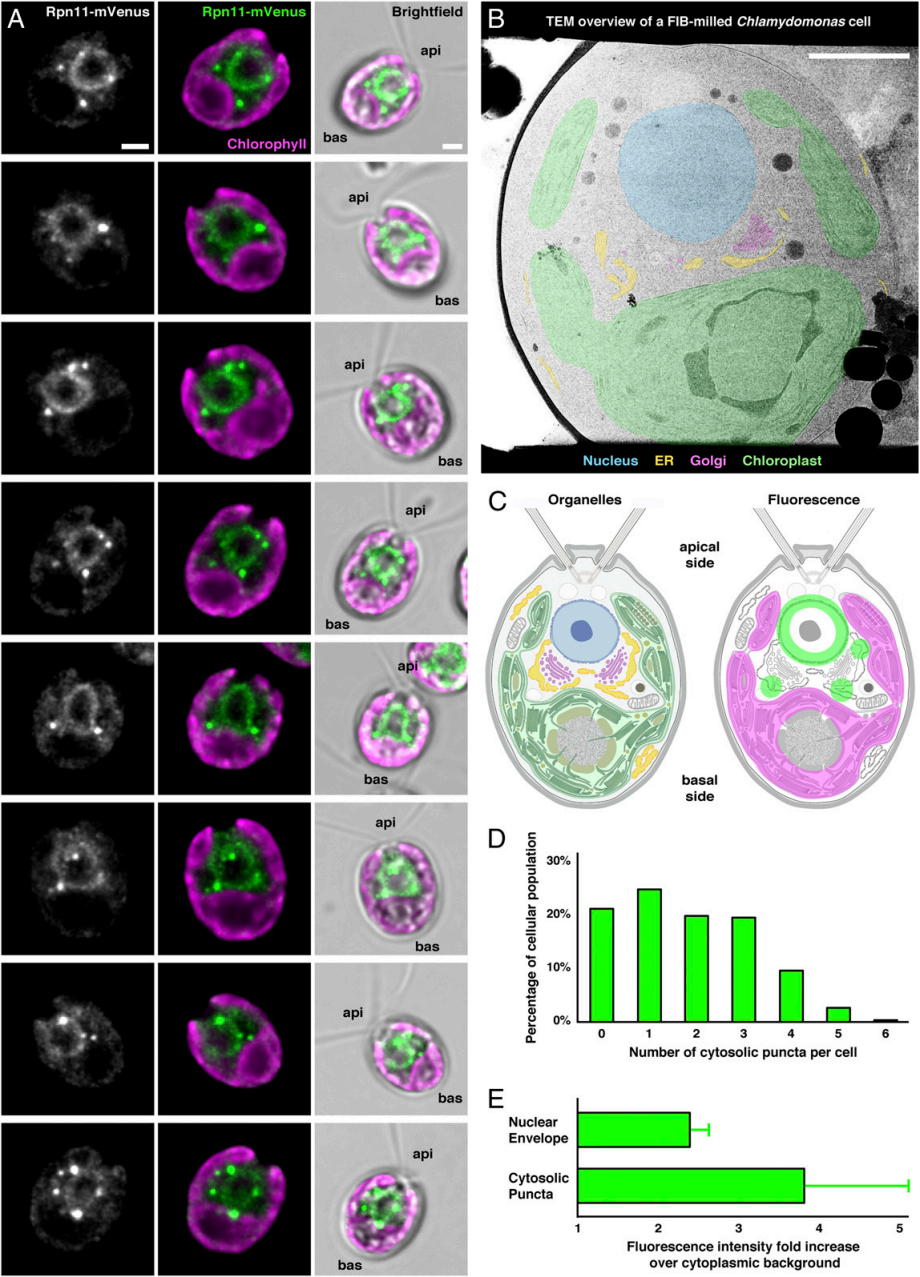
In *Chlamydomonas* cells, proteasomes are concentrated at the nuclear envelope and in cytosolic puncta. (*A*) Live *Chlamydomonas mat3-4* cells expressing the tagged proteasome subunit Rpn11-mVenus, imaged in 3D by wide-field deconvolution fluorescence microscopy. *Left* column: maximum-intensity projection of Rpn11-mVenus, showing localization to the nuclear envelope and cytosolic puncta. *Middle* column: Rpn11-mVenus (green) overlaid with chlorophyll autofluorescence (magenta). *Right* column: both fluorescence signals overlaid on a bright-field image, with protruding flagella distinguishing the apical (api) and basal (bas) sides of the cell. (*B*) Transmission electron microscopy overview image of a *Chlamydomonas* cell thinned to ∼200 nm with a cryo-FIB. The nucleus, ER, Golgi, and chloroplast are pseudocolored as indicated. (*C*) Diagram of *Chlamydomonas* organelle architecture (*Left*; colored as in *B*) and fluorescence localization from *A* (*Right*). The Rpn11-mVenus puncta are predominantly localized to the cytoplasm between the nucleus and the chloroplast, a region occupied by ER and Golgi. (*D*) Histogram of the number of cytosolic puncta per cell. *N* = 565 cells. (*E*) The intensity of Rpn11-mVenus fluorescence at the nuclear envelope and within the cytosolic puncta, normalized by fold change over each cell’s cytosolic background. Puncta have nearly twice the intensity of the nuclear envelope, indicating higher proteasome concentration. Error bars show SD. (Scale bars: 2 μm in *A* and *B*.)

To get an overview of proteasome localization within *Chlamydomonas* cells, we expressed the proteasome subunit Rpn11 fused with the fluorescent protein mVenus, and then examined live cells in three dimensions (3D) by wide-field deconvolution microscopy. As expected from our previous cryo-ET discovery that proteasomes tether to NPCs (5), we observed a clear fluorescence signal along the nuclear envelope (Fig. 1*A*). This localization confirms that the Rpn11-mVenus protein was incorporated into functional proteasomes. In addition, bright puncta could be seen in the cytoplasm adjacent to the nucleus. These puncta were most commonly observed between the basal side of the nucleus and the concave inner surface of the cup-shaped chloroplast, a region of the cytoplasm occupied by abundant ER and Golgi (Fig. 1 *B* and *C*). Cytosolic puncta were observed in 78% of the population; cells most frequently contained 0 to 3 puncta, with up to 6 puncta per cell (Fig. 1*D*). The Rpn11-mVenus signal at the nuclear envelope was ∼2.4-fold more intense than the cytoplasmic background signal, whereas puncta intensity was more variable but on average ∼3.9-fold more intense than the background, indicating a higher concentration of proteasomes in the puncta than at the nuclear envelope (Fig. 1*E*).

Tracking the cytosolic puncta over time revealed that these structures are dynamic. We recorded time series of living Rpn11-mVenus cells with 3D *Z* stacks acquired once per minute. Despite fairly rapid photobleaching, we observed the de novo assembly of new cytosolic puncta in multiple cells during the 14-min time series (*SI Appendix*, Fig. S3 and Movie S1). The puncta often appeared to assemble adjacent to the nuclear envelope and then migrate outward toward the ER (*SI Appendix*, Fig. S3 *B*–*D*). Even more striking, the cytosolic puncta appeared to fuse with each other (*SI Appendix*, Fig. S4 and Movie S2). Before fusion, two puncta often exhibited coupled motion (for example, see *SI Appendix*, Fig. S4*D*). Immediately following fusion, the resulting single punctum increased in fluorescence intensity, reflecting the accumulation of Rpn11-mVenus from the two fused puncta within a diffraction-limited spot. These puncta were observed to remain as single entities for several minutes after fusion (up to 9 min of the 14-min time series; see *SI Appendix*, Fig. S4*C*), suggesting the events were bona fide fusion.

**Fig. 2.**
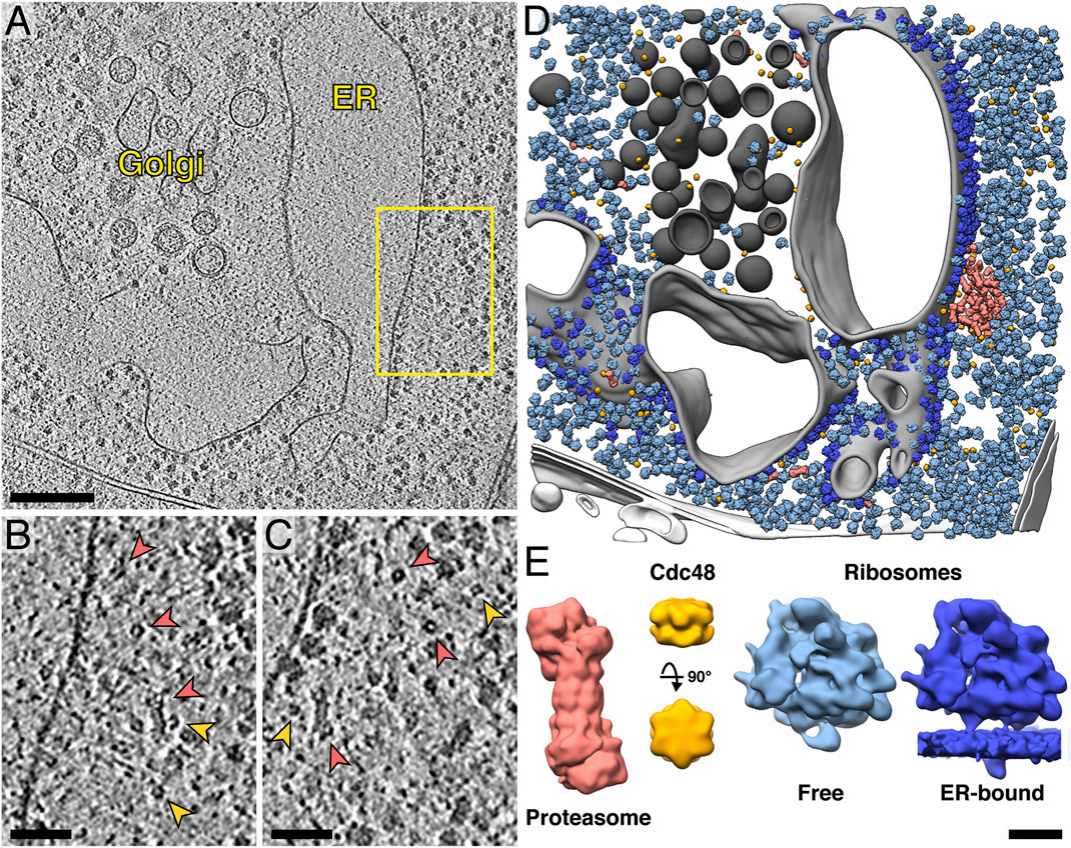
Imaged with in situ cryo-ET, cytosolic proteasomes cluster at the ER membrane. (*A*) Slice through a tomogram that targets the cytoplasm of a *Chlamydomonas mat3-4* cell, showing the ER and the Golgi. (*B* and *C*) Two different *Z* slices through the proteasome cluster boxed in *A*. Red arrows, proteasomes; yellow arrows, Cdc48 (Movie S4). (*D*) Corresponding segmentation (Golgi, dark gray; ER, light gray; other organelles, white) with in situ subtomogram averages of proteasomes (red, 19.5-Å resolution), Cdc48 (yellow, 32.8-Å resolution), free cytosolic ribosomes (light blue, 18.8-Å resolution), and membrane-bound ribosomes (dark blue, 21.9-Å resolution) mapped back into the cellular volume. (*E*) Enlarged views of the in situ subtomogram averages. (Scale bars: 200 nm in *A*, 50 nm in *B* and *C*, and 10 nm in *E*.)

To investigate the molecular architecture of these cytosolic proteasome foci, we visualized the native cellular environment in 3D by in situ cryo-ET (29). Because the small size and transient nature of the fluorescent puncta made direct correlation between fluorescence and cryo-ET unreliable, we instead examined the distribution of proteasomes in nontransgenic *Chlamydomonas* cells. Our search was aided by the exceptionally reproducible cellular architecture of these algae, which restricted the proteasome foci to a region around the nucleus that is rich in ER and Golgi (Fig. 1 *B* and *C*). In 76 cellular tomograms covering both the cytoplasm and nuclear periphery, cytosolic 26S proteasomes were identified via template matching and subtomogram averaging, as detailed in ref. 5. Mapping the proteasomes back into the cellular environment revealed densely packed clusters of proteasomes adjacent to the ER membrane (Fig. 2, *SI Appendix*, Figs. S5–S7, and Movies S3 and S4). The clusters were located near the ribosome-bound surface of the rough ER, away from the ER’s specialized ribosome-free region that serves as an exit site for COPII-mediated transport to the Golgi (Fig. 2*D*). Comparing the proteasome positions to simulated random distributions of proteasomes within the same cellular volumes confirmed the clustering behavior (*SI Appendix*, Fig. S8*A*). Six clusters were distinguished from the rest of the cytosolic proteasome population by *k*-means analysis, with 19 to 42 proteasomes per cluster.

**Fig. 3.**
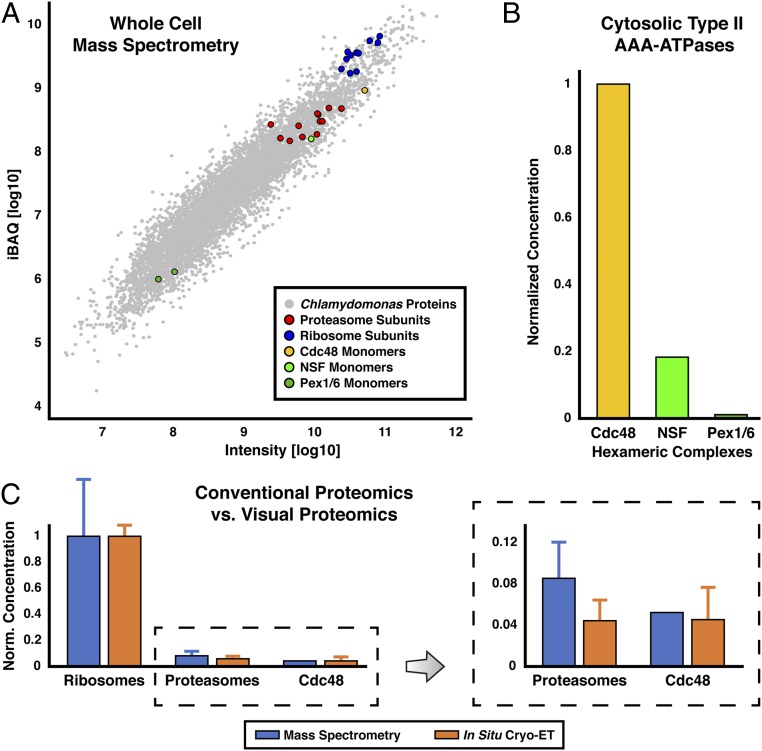
Whole-cell mass spectrometry shows that Cdc48 is the most abundant type II AAA-ATPase and confirms the relative abundance of complexes identified by in situ cryo-ET. (*A*) Scatter plot of the proteome from *Chlamydomonas mat3-4* cells (the same strain used for cryo-ET). Protein abundance is plotted as intensity versus the iBAQ value (intensity-based absolute quantification; raw protein intensity divided by the number of peptides) (99). Measured proteasome, ribosome, Cdc48, NSF, and Pex1/6 subunits are marked with red, blue, yellow, light green, and dark green circles, respectively. Note that because Cdc48 and NSF form homo-hexamers, the number of macromolecular complexes is 6-fold lower than the protein abundance. Pex1 and Pex6 form a hetero-hexamer, so the number of macromolecular complexes is 3-fold lower than the protein abundance. (*B*) The relative levels of 3 common cytosolic type II AAA-ATPases from the whole-cell proteomics, normalized to Cdc48. (*C*) The relative levels of ribosome, proteasome, and Cdc48 complexes from the proteomics (blue) and cryo-ET (orange). Error bars show SD (between subunits for the mass spectrometry and between tomograms for the cryo-ET). Concentrations are normalized by the ribosome levels. The plot on the *Right* shows a zoom-in on the proteasomes and Cdc48 to more clearly display their relative abundance. The slightly lower concentration of proteasomes determined by cryo-ET is likely due to the high abundance of proteasomes in the nucleus (5); in this analysis, only cytosolic proteasomes were quantified by cryo-ET, whereas whole cells were measured by mass spectrometry.

Next, we sought to identify a second component of the ubiquitin–proteasome system, the AAA-ATPase Cdc48 (homolog of mammalian p97). Template matching and subtomogram averaging yielded a hexameric structure for Cdc48 (Fig. 2*E* and *SI Appendix*, Fig. S9*C*) that resembles the single-particle cryo-EM structure (30, 31). Due to the small size of this 540-kDa complex, we took several measures to ensure correct and comprehensive identification. First, we thoroughly classified our candidate Cdc48 subvolumes to discard false positives from the average (*SI Appendix*, Fig. S10). Next, we successfully calculated reference-free averages, both with and without imposed 6-fold symmetry, confirming that the subvolumes could reach a Cdc48 structure without directing the alignment toward an initial reference (*SI Appendix*, Figs. S9*A* and S11*A*) (32, 33). Even without imposing symmetry during alignment, the resulting average shows clear characteristics of a hexamer (*SI Appendix*, Fig. S11). This reference- and symmetry-free average resembles a mixture of both ring (30, 31) and staircase (34) conformational states (*SI Appendix*, Fig. S12). However, the small size of Cdc48 prevented classification from distinguishing these conformations, and thus, we were unable to separate the complexes into subpopulations. Finally, we employed whole-cell mass spectrometry to verify that there is a high concentration of Cdc48 in our *Chlamydomonas* strain (Fig. 3) (35). Cdc48 and proteasomes were detected with similar protein abundance, in agreement with the distribution of the two complexes in our tomograms (Fig. 3*C*). Furthermore, Cdc48 was far more abundant than the other common cytosolic type II AAA-ATPases, NSF and Pex1/6 (Fig. 3*B*) (36). Thus, even if our structural analysis was unable to distinguish between different species of type II AAA-ATPases, the percentage of incorrectly assigned Cdc48 particles would be low. In addition to Cdc48, we also generated subtomogram averages of both free and membrane-bound 80S ribosomes (Fig. 2*E* and *SI Appendix*, Fig. S9*D*), as described in ref. 37.

**Fig. 4.**
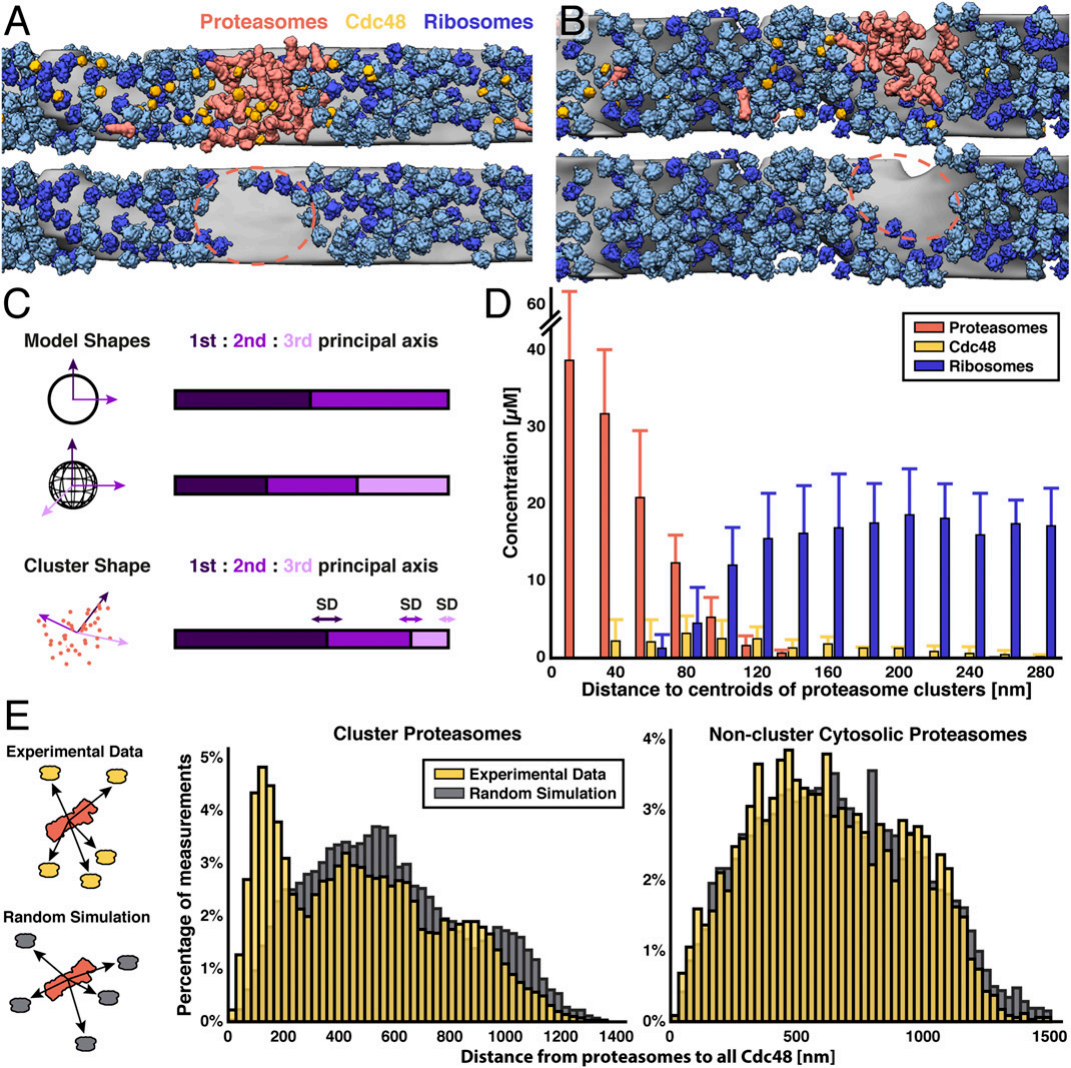
Proteasomes and Cdc48 form globular ribosome-excluding microcompartments. (*A* and *B*) Two close-up views of proteasome clusters within the cell, looking toward the ER membrane (gray) from the cytosol. *Top* image: view displaying proteasomes (red), Cdc48 (yellow), and ribosomes (membrane-bound, dark blue; free, light blue). *Bottom* image: the same view with only the ribosomes displayed, revealing that ribosomes are excluded from the proteasome cluster and adjacent patch on the ER membrane (red dashed line). (*C*) PCA of proteasome cluster shape. *Left* side: eigenvectors (3 shades of purple) are drawn for a model circle and sphere (*Top* 2 rows), as well as an example cluster (*Bottom* row, red dots are proteasome center positions). *Right* side: the corresponding ratio of eigenvalues for each shape, with SDs displayed as bidirectional arrows. *N* = 6 proteasome clusters (*SI Appendix*, Figs. S6 and S7). (*D*) Radial concentrations of proteasomes, Cdc48, and ribosomes outward from the centroid positions of the proteasome clusters reveal a cellular microcompartment of distinct composition. Proteasomes are strongly accumulated in the microcompartment. Cdc48 are found throughout the cytosol but peak at the microcompartment border. Ribosomes are only found beyond the microcompartment border, reaching a constant concentration throughout the cytosol. The fairly broad transition from proteasomes to ribosomes at the border is primarily due to combining microcompartments of different sizes (range of diameters on long axis: 157 to 235 nm) for this analysis. Error bars show SD. (*E*) Distances from every proteasome to every cytosolic Cdc48, performed separately for cluster proteasomes (*Left* plot, yellow) and noncluster proteasomes (*Right* plot, yellow). The analysis was repeated on simulated data where the same number of Cdc48 complexes was randomly placed into the same cellular volumes (gray). The experimental data for the cluster proteasomes shows a nonrandom peak at <200 nm, indicating that Cdc48 clusters together with proteasomes in the microcompartments.

Mapping the proteasomes, ribosomes, and Cdc48 structures back into the cellular environment enabled us to draw several conclusions. The proteasome clusters exclude ribosomes, both in the cytosol as well as on the ER membrane; there are small ribosome-void patches exactly at the spots where clusters touch the membrane (Fig. 4 *A* and *B* and *SI Appendix*, Fig. S7). Principal-component analysis (PCA) of all of the proteasome clusters in our dataset revealed a slightly flattened, globular-shaped cloud with a diameter of 210 ± 30 nm (first PCA axis) by 101 ± 29 nm (second PCA axis) (Fig. 4*C*). Based on their shape, we determined the centroid of each cluster and evaluated the radial protein concentration from the centroid for proteasomes, Cdc48, and ribosomes (Fig. 4*D*). The 30 to 40 μM concentration of densely packed proteasomes at the cluster center is higher than the ∼8 μM proteasome concentration we previously measured at the nuclear envelope (5), consistent with our fluorescence intensity measurements (Fig. 1*E*). We observed a clear separation between proteasomes and ribosomes, with the latter’s concentration increasing right at the cluster border to a constant cytosolic level. Cdc48, however, was found throughout the proteasome cluster and surrounding ribosome region, but its concentration peaked at the periphery of the cluster, with a ∼5-fold increase compared to the rest of the cytosol. To show that Cdc48 not only colocalizes with the proteasome clusters but also clusters with them statistically, we calculated the distances from each proteasome to each Cdc48 within the tomograms (Fig. 4*E*). This analysis was performed independently for proteasomes inside and outside the clusters, both for the experimental data and for simulated data where the same number of Cdc48 complexes were placed randomly throughout the same cytosolic volumes. Whereas the distance distribution of nonclustered cytosolic proteasomes to Cdc48 appeared random (Fig. 4*E*, *Right*), there was a clear nonrandom peak at short distances between clustered proteasomes and Cdc48 (Fig. 4*E*, *Left*), indicating an accumulation of Cdc48 at the border of the proteasome cluster. In agreement with this finding, a Kolmogorov–Smirnov test showed with high statistical significance that the distances between clustered proteasomes and their nearest Cdc48 neighbors were nonrandom (*SI Appendix*, Fig. S8*B*). In contrast, distance analysis between proteasomes and ribosomes revealed no correlation (*SI Appendix*, Fig. S8*E*). Taking these findings together, the proteasome clusters define cytosolic microcompartments of distinct protein composition, excluding ribosomes while concentrating proteasomes and Cdc48 together at a specialized patch of the ER membrane.

**Fig. 5.**
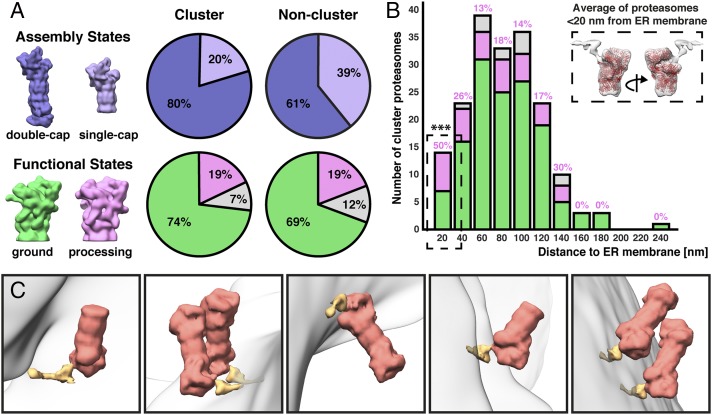
Analysis of proteasome states within degradation microcompartments. (*A*) Comparison of assembly state and functional state frequencies between the cluster and noncluster cytosolic proteasomes. Cluster proteasomes have a higher percentage of double-capped (80%) compared to the cytosolic population (61%). For both populations, the majority of proteasome caps are in the ground state (69 to 74%). Double-capped, purple; single-capped, lavender; ground state, green; substrate-processing state, pink; unclassified, gray. (*B*) Functional states of cluster proteasome caps as a function of distance to the ER membrane. The percentages of processing caps are written in pink over each bar in the graph. Proteasomes <20 nm from ER have a significantly higher fraction of processing caps compared to the rest of the cluster proteasomes (for statistical test, see *Methods*). Subtomogram averaging of these ER-proximal proteasome caps reveals an extra density that likely corresponds to engaged substrate (dashed *Inset*, red: fitted proteasome molecular structure; see *SI Appendix*, Fig. S13 for evidence that the extra density is bound at the proteasome’s substrate engagement site). (*C*) Mapping each of the ER-proximal processing state proteasomes (red) back into the cellular volumes reveals that the extra density (orange) always connects to the ER membrane (gray), consistent with a putative substrate.

To gain more insight into the function of these ER-associated microcompartments, we used classification to take a closer look at the structures of the proteasomes contained within. Classification can distinguish several features of proteasomes in situ, including whether the 20S core particle is capped by one or two 19S regulatory particles, and whether each 19S cap is in an inactive ground state or an active substrate-processing state (5, 38, 39). Our resolution was sufficient to clearly distinguish these structural differences for each proteasome. Interestingly, we saw an increased percentage of double-capped proteasomes within the clusters (80%) compared to the rest of the cytosol (61%) (Fig. 5*A*, *Top*). Such an increased frequency of double-capped proteasomes indicates a higher degradation capacity and was recently observed for accumulated proteasomes within neurodegenerative aggregates (39). In contrast, the proteasome caps showed very similar substrate-processing state frequencies (19%) inside and outside the clusters (Fig. 5*A*, *Bottom*). While this demonstrates that the proteasomes within the microcompartments are active, we further dissected the functional states of these clustered proteasomes by analyzing the frequency of substrate-processing 19S caps as a function of distance from the ER membrane (Fig. 5*B*). Immediately adjacent to the ER (<20 nm), we observed a statistically significant higher percentage of substrate-processing caps (50%). Averaging these ER-proximal proteasomes revealed an extra density attached to their substrate-processing caps (Fig. 5*B*, *Inset*). In a previous in situ cryo-ET study by Guo et al. (39), proteasomes with substrate-processing caps were observed to bind poly-GA neurodegenerative aggregates. The subtomogram average of these proteasomes showed a large extra density bound to the caps that clearly originated from the engaged aggregate. As the 19S caps were in the substrate-processing state, and the gate to the 20S proteolytic chamber was open, the extra density could be definitively assigned to substrate that is being fed into the proteasome. Comparing the structure from Guo et al. with our subtomogram average of ER-proximal proteasomes revealed a clear overlap of the extra densities at the proteasome’s substrate-engagement site near Rpn1, indicating that the extra density we observe is likely substrate (*SI Appendix*, Fig. S13). Upon mapping the proteasomes and putative substrate densities back into the cellular volumes, we found that the substrate densities always originated from the ER membrane (Fig. 5*C*). Thus, proteasomes inside the microcompartments are not only active, but their level of activity is correlated with their proximity to the ER membrane, with the closest proteasomes apparently pulling proteins directly out of the membrane.

To further investigate the function of these ER-associated proteasome clusters, we monitored the effects of pharmacological treatments on the proteasome puncta seen by fluorescence microscopy (*SI Appendix*, Fig. S14). Treatment with the ER stress-inducing drug tunicamycin had little effect on proteasome localization but moderately decreased the number of cytosolic puncta. One interpretation could be that the proteasome puncta do not respond to the acute misfolding of ER proteins but rather perform a constitutive degradation function at the ER membrane. However, because many of the cytosolic proteasomes were already clustered within puncta before adding the drug, it is hard to conclude much from the observation that additional puncta do not form. Treatment with the proteasome inhibitor MG132 (40) also reduced the number of cytosolic puncta and caused a dramatic accumulation of bright proteasome puncta at the nuclear envelope. This striking redistribution between proteasome populations warrants future study and may prove useful for dissecting the function of NPC-tethered proteasomes. Treatment with the Cdc48 inhibitor NMS-132 (41) appeared to be toxic to *Chlamydomonas* cells and resulted in diffuse cytosolic proteasome signal.

## Discussion

**Fig. 6.**
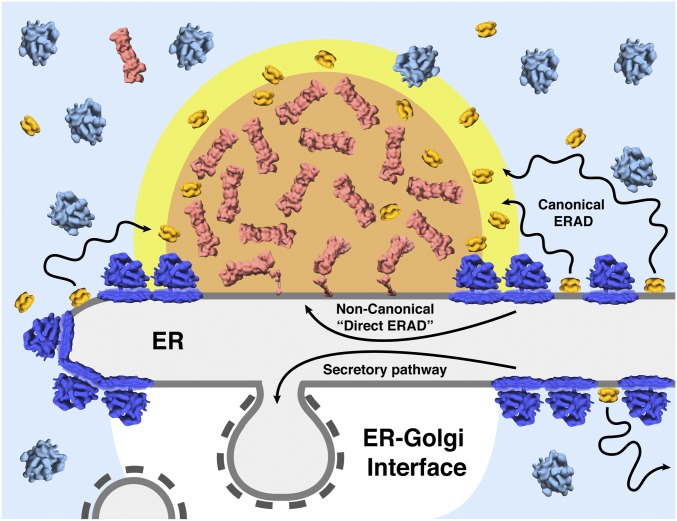
Molecular architecture of non–membrane-bound degradation microcompartments at the ER. Proteasomes (red) and Cdc48 (yellow) cluster together to form concentrated ∼200-nm microcompartments that exclude cytosolic ribosomes (light blue) (Fig. 4 and *SI Appendix*, Figs. S6 and S7). These microcompartments directly contact small patches on the rough ER membrane (gray) that are devoid of membrane-bound ribosomes (dark blue) and may be enriched in ERAD substrates. The proteasomes closest to the membrane have regulatory caps that are in the substrate-processing conformation and are engaged at their substrate-binding sites with densities emanating from the ER (Fig. 5 and *SI Appendix*, Fig. S13). Thus, these densities are likely substrates that are undergoing removal from the ER directly by proteasomes (a noncanonical pathway we term “direct ERAD”). Cdc48 complexes are enriched at the microcompartment periphery, where they may be handing substrates to the proteasomes in the final step of the canonical ERAD pathway. Unlike proteasomes, Cdc48 is commonly found all along the ER membrane (82% of ER-proximal proteasomes, but only 20% of ER-proximal Cdc48, are localized to the microcompartments). Therefore, Cdc48 might extract substrates from the ER, then diffuse through the cytosol until it encounters a proteasome, the majority of which are clustered in microcompartments. Cytosolic diffusion of Cdc48, followed by substrate hand-off to proteasomes, could explain the peripheral localization of Cdc48 around the proteasome clusters. The microcompartments may additionally serve as degradation centers for cytosolic proteins. The microcompartments are positioned away from the larger ribosome-free region of the ER where COPII-coated vesicles bud en route to the Golgi. This spatially segregates the ERAD and secretory pathway machinery, and may aid in sorting ER proteins between trafficking and degradation.

Combining live-cell fluorescence microscopy with in situ cryo-ET, we observed that a significant fraction of cytosolic protein degradation in *Chlamydomonas* is likely carried out within dynamic, non–membrane-bound microcompartments that have their own characteristic architecture (Fig. 6). These globular microcompartments exclude ribosomes and consist of an inner core of active proteasomes surrounded by a loose shell of Cdc48 (Fig. 4). Each microcompartment is positioned directly adjacent to a specialized patch on the ER membrane that lacks membrane-bound ribosomes. While our imaging approach could only clearly identify the large cytosolic proteasome and Cdc48 complexes, it seems plausible that the adjacent ribosome-free patch on the ER membrane also has a distinct molecular composition. This small membrane region, which matches the dimensions of the cytosolic microcompartment (Fig. 4 *A* and *B* and *SI Appendix*, Fig. S7), may be enriched with both ERAD substrates and ER-resident components of the ERAD pathway, such as E3 ligases and retrotranslocation channels. By concentrating the cytosolic and membrane-embedded ERAD machinery together into a few degradation hot spots along the ER membrane, the microcompartments could greatly increase ERAD efficiency. The degradation microcompartments are localized away from the ER–Golgi interface, a larger zone of ribosome exclusion that is enriched with mediators of vesicular trafficking, such as coat proteins (27, 42). This segregation of COPII budding and ERAD to different regions of the ER may facilitate the sorting of ER proteins either for onward traffic through the secretory pathway or for removal and degradation (Fig. 6). The close association of degradation microcompartments to the ER membrane may also explain the coupled motion we observed before two microcompartments fuse (*SI Appendix*, Fig. S4 and Movie S2). These microcompartments are likely bound to the same membrane, which would enable them to readily encounter each other and fuse.

One surprising observation is the direct engagement of proteasomes with density that emanates from the ER membrane (Fig. 5*C*). Unlike NPC-tethered proteasomes, which are bound by a tethering density at the Rpn9 subunit of their 19S cap (5), these proteasomes bind the ER-emanating density on the opposite side of their cap, near Rpn1. Two previous in situ studies have observed putative substrate occupying this specific region (38, 39). In particular, the study by Guo et al. clearly shows that proteasomes with caps in the substrate-processing conformational state bind neurodegenerative aggregates at this Rpn1-adjacent region. Like the aggregate-engaged proteasomes in Guo et al., the ER-engaged proteasomes observed in our study are also in the substrate-processing state and are also bound to density at the Rpt AAA-ATPase ring near Rpn1 (Fig. 5*B* and *SI Appendix*, Fig. S13). Based on this structural evidence, we conclude that the density attaching these proteasomes to the ER is likely substrate that the proteasomes are directly extracting from the membrane. Due to the direct action of proteasomes on ER proteins, we term this mechanism noncanonical “direct ERAD” (Fig. 6). In support of this mechanism, there is evidence that the proteasome’s Rpt AAA-ATPase can extract specific substrates from the ER (43–45). However, it remains to be studied whether the direct ERAD we observe is specific for a subset of ER-localized substrates. The rate-limiting step for proteasome-mediated degradation is the mechanical unfolding of substrates by the proteasome’s Rpt AAA-ATPase, which could take minutes for well-folded substrates (46). Such a long residency time of proteasomes engaged with ER substrates would explain why we captured several of these events in our tomograms.

Cdc48 is canonically described to function upstream of the proteasome in the ERAD pathway, extracting misfolded proteins from the ER membrane and delivering them to cytosolic proteasomes for degradation (17, 18, 47). In the degradation microcompartments, we observed a loosely organized cloud of Cdc48 complexes around the cytosolic periphery of the proteasome cluster instead of at the interface between the proteasomes and the ER membrane (Fig. 4*D*, *SI Appendix*, Fig. S8*C*, and Movies S3 and S4). While at first glance this observation might seem to be inconsistent with canonical Cdc48-mediated ERAD, quantifying the distribution of proteasomes and Cdc48 along the whole ER surface reveals a more compatible explanation: Of the complexes within 200 nm of the ER membrane, 82% of the proteasomes but only 20% of Cdc48 are found inside the microcompartments. It is likely that the other 80% of ER-proximal Cdc48 performs canonical ERAD, segregating substrates from Hrd1 at dispersed locations along the ER membrane and then diffusing through the cytoplasm until the Cdc48 encounters a proteasome to degrade its substrate. As the majority of ER-proximal proteasomes are clustered within the microcompartments, diffusing Cdc48 would accumulate at the microcompartment periphery to hand substrates to the proteasomes, completing the canonical ERAD pathway (Fig. 6). The ATPase rate of Cdc48 is about 10 times faster than the proteasome (46, 48), which suggests that Cdc48 may have a shorter residency time at the ER. This would explain why we do not observe accumulation of Cdc48 at the ER membrane (*SI Appendix*, Fig. S8*D*). Given the high concentration of proteasomes in the microcompartments, these regions may additionally serve as hubs for the degradation of cytosolic proteins. Indeed, quality control of cytosolic proteins has been shown to require ER-resident ubiquitin ligases (49, 50), and thus may also occur at the surface of the ER membrane.

Recent in vitro work suggests that Cdc48 may dock onto the proteasome’s 20S core in place of the 19S regulatory cap (51). To look for this interaction, we searched the tomograms with a hybrid Cdc48-proteasome template structure (*SI Appendix*, Fig. S15). Reference-free alignment of the top hits produced a normal 26S proteasome structure, indicating that Cdc48-proteasomes are not present in significant numbers within the cellular volumes. Indeed, even though our approach attempted to bias the template matching toward a Cdc48-proteasome hybrid structure, we nonetheless could only recover normal 26S proteasomes.

One question that remains for future investigation is the prevalence of degradation microcompartments in other organisms. Much of the canonical ERAD pathway was elucidated through extensive study of the budding yeast *Saccharomyces cerevisiae* (10, 13, 16, 47, 52–55). Although most of the ERAD machinery is conserved between yeast and *Chlamydomonas* (*SI Appendix*, Figs. S1 and S2), the cellular architecture of the secretory system is not. *Chlamydomonas* cells have an elaborate and highly organized ER and Golgi, with a clearly defined interface between the two organelles (26–28, 37). In contrast, the ER and Golgi of *S. cerevisiae* have simplified architecture; the Golgi is not stacked and appears to be randomly positioned relative to the ER, lacking the robust interface region found in *Chlamydomonas* and mammalian cells (56, 57). Thus, *S. cerevisiae* may lack this separation of protein degradation in microcompartments away from the secretory traffic of the ER–Golgi interface (Fig. 6).

How do the degradation microcompartments form, and how are they anchored to the ER membrane? Some answers may be found in the composition of the ribosome-free patch of membrane adjacent to the microcompartment, which may contain proteins that help recruit proteasomes to the ER. It will be interesting to investigate the parallels between this noncanonical direct ERAD mechanism and canonical ERAD, where Cdc48 recruitment and substrate engagement are mediated by Ubx2 and Hrd1, respectively (13, 15, 54). As we only observed proteasome clusters in contact with the ER membrane and not free-floating in the cytosol, recruitment of proteasomes to the ER may nucleate proteasome clustering.

Unlike the proteasomes tethered to NPCs that we observed in an earlier study (5), the proteasomes within the microcompartments are randomly oriented. Instead, the degradation microcompartments seem to be more architecturally similar to proteasome storage granules (PSGs), spherical non–membrane-bound proteasome clusters that form in the cytosol of quiescent cells (58). PSGs are proposed to form by liquid–liquid phase separation (59), but the multivalent “molecular glue” that drives PSG condensation has not been identified. Proteomic analysis of isolated PSGs found that these compartments are primarily composed of proteasomes and monoubiquitin (60). Whether monoubiquitin can mediate the phase separation of proteasomes remains to be tested. Unlike the degradation microcompartments described in our study, PSGs are proposed to store inactive proteasomes. It will be interesting to determine whether these two non–membrane-bound compartments, which perform different functions, share a common mechanism of proteasome clustering.

Several properties of the degradation microcompartments are consistent with liquid–liquid phase separation. The microcompartments concentrate specific components (proteasomes and Cdc48) while excluding others (ribosomes) (Fig. 4). The microcompartments are spherical at the resolution of light microscopy (Fig. 1 and *SI Appendix*, Figs. S3 and S4), and the dense but disorganized packing of proteasomes (*SI Appendix*, Fig. S7 and Movie S3) is reminiscent of the packing of Rubisco within the liquid-like pyrenoid (61). Furthermore, the microcompartments appear to readily fuse with each other upon contact (*SI Appendix*, Fig. S4 and Movie S2). Fusion has been described for a variety of liquid-like compartments, including P-granules (62), stress granules (63), nucleoli (64), and heterochromatin (65). The ability of two compartments to fuse is a hallmark of liquid–liquid phase separation and distinguishes a liquid from a gel (66). Additional evidence for a liquid phase can be established by observing internal mixing after photobleaching half of a phase-separated compartment (61, 62, 65). However, this approach may not be feasible for the degradation microcompartments due to their small ∼200-nm size. During the activation of transmembrane receptors, including nephrin and T cell receptor, the clustering of membrane proteins into domains spanning hundreds of nanometers is coupled to the liquid–liquid phase separation of their cytosolic binding partners (67, 68). It is believed that coupled phase separation between the membrane and cytoplasm may represent a general principle in cellular organization (69). We propose that similar forces drive the clustering of membrane proteins within distinct ER domains that contact the cytosolic microcompartments of proteasomes and Cdc48.

## Methods

### Cell Culture.

For fluorescence imaging, proteomics, and in situ cryo-ET, we used *mat3-4* (strain CC-3994) (70) *Chlamydomonas reinhardtii* cells, provided by the *Chlamydomonas* Resource Center (University Minnesota, Minneapolis, MN). This strain has smaller cells, which greatly improves vitrification by plunge freezing. Cells were grown in Tris–acetate–phosphate (TAP) medium under constant light conditions (∼90 µmol photons⋅m^−2^⋅s^−1^) and normal atmosphere. Cells were harvested when they reached midlog phase.

### Expression of Fluorescently Tagged Rpn11 in *Chlamydomonas*.

We chose to fluorescently tag the proteasome cap’s Rpn11 subunit because it has previously been GFP-tagged without discernable impacts on cellular function in both budding yeast and fission yeast (71). We cloned into the pLM005 vector because this tool has successfully been used to localize over 100 different proteins in *Chlamydomonas* cells to produce a spatial interactome (72). The pLM005-Rpn11 construct (Rpn11 tagged at its C terminus with mVenus-3xFLAG) was generated by PCR amplification of the full-length RPN11 sequence from purified genomic DNA (forward primer, 5′-GCTACTCACAACAAGCCCAGTTATGGACGGCTTGCAGCGCATGTT-3′; reverse primer, 5′-GAGCCACCCAGATCTCCGTTGAAGACCACCGTGTCCAGCATGG-3′). The PCR product was then subcloned into the pLM005 vector (*Chlamydomonas* Resource Center, University of Minnesota) (72) using a Gibson assembly kit (NEB) according to the manufacturer’s instructions. The plasmid was sequenced to confirm correct insertion of the RPN11 gene (pLM005 forward primer, oMJ237, 5′-GGAGGTACGACCGAGATGGCT-3′; pLM005 reverse primer, oMJ555, 5′-CACGTCGCCGTCCAGCTC-3′). Following a transformation protocol adapted from Zhang et al. (73), the pLM005-RPN11 plasmid was linearized with the DraI restriction enzyme (NEB) and electroporated into *mat3-4* cells. Transformants were screened on TAP plates supplemented with 20 μg/mL paromomycin and verified by Western blot using an antibody against the FLAG tag. To improve Rpn11-mVenus expression, we reselected the same transformants using TAP plates containing 20 μg/mL paromomycin, and then grew the cells in liquid TAP media containing 20 μg/mL paromomycin prior to imaging. Rpn11-mVenus expression did not affect the growth rate of the cells. The nuclear localization of Rpn11-mVenus (Fig. 1*A*) indicates that this transgenically expressed protein was incorporated into functional proteasomes, as this localization has previously been described by in situ cryo-ET (5).

### Live-Cell Fluorescence Imaging.

Cells from log-phase cultures were immobilized for 15 min on glass bottom u-Slide 8-well micro plates (Ibidi) that were coated with 5 mM poly-l-lysine. Excess culture was washed away with TAP medium. For the inhibitor experiments (*SI Appendix*, Fig. S14), single liquid cultures were split into batches that were treated with either 50 μM MG132 (Sigma-Aldrich; 474787), 5 μg/mL tunicamycin (Sigma-Aldrich; T7765), or 5 μg/mL NMS-873 (Sigma-Aldrich; SML1128) for 2 h. During the final 10 min, cells were immobilized on slides and then washed with TAP media containing the same concentration of inhibitor. All inhibitor tests were performed twice, each time with a freshly grown culture.

Imaging was performed on a Leica DMI6000 B inverted microscope, equipped a with coolLED pe-4000 LED source and a Leica DFC9000 GT sCMOS camera, and operated with LAS X software (Leica Microsystems). The *Z* stacks in Fig. 1 were acquired using an HCX PL APO 63×/1.20 numerical aperture (N.A.) water-immersion objective, whereas the time series in *SI Appendix*, Figs. S3 and S4 (Movies S1 and S2) were acquired with a 63×/1.4 N.A. oil-immersion objective and a hardware infrared autofocus system. The following fluorescence settings were used: mVenus, 500-nm excitation, 535/30-nm emission filter; chlorophyll autofluorescence, 635-nm excitation, 680/40-nm emission filter. For the higher-fidelity imaging in Fig. 1, we acquired *Z* stacks composed of 38 to 48 slices 0.3 μm apart, covering much more than the full cellular volume to aid deconvolution. For the time series in *SI Appendix*, Figs. S3 and S4, we acquired *Z* stacks of 20 slices, 0.4 μm apart, every 1 min for 15 cycles. These parameters were selected as a balance between covering the full cellular volume and minimizing photobleaching. The data were acquired in 4 imaging sessions: 2 for the quantification of puncta (Fig. 1) and 2 for the time series (*SI Appendix*, Figs. S3 and S4).

### Fluorescence Image Analysis.

*Z* stacks were deconvolved using Huygens Essential software (Scientific Volume Imaging). All further image processing and analysis were performed with Fiji software (74). Cells and cytosolic puncta were manually counted using entire *Z* stacks. Fluorescence intensity of the nuclear envelope was calculated by averaging the signal of the mVenus channel from 2 nonoverlapping regions of interest (ROIs) drawn on the center slice of the nuclear envelope. Similarly, the average intensity of the center 4 pixels was measured individually for every punctum using the signal from a single slice. For both the nuclear envelope and cytosolic puncta, fluorescence intensity was internally normalized to the intensity of the mVenus background signal measured by an ROI drawn in the cytosol of each cell.

### Cell Vitrification and Cryo-FIB Milling.

Both vitrification and FIB milling protocols were performed as previously reported (23, 75). Using a Vitrobot Mark 4 (FEI Thermo Fisher), the cell culture (4 µL of ∼1,000 cells per µL) was blotted onto R2/1 carbon-coated 200-mesh copper EM grids (Quantifoil Micro Tools) and plunge-frozen in a liquid ethane/propane mixture. Cryo-FIB sample preparation was performed in either a Scios or Quanta dual-beam FIB/SEM instrument (FEI Thermo Fisher). Mounted in an Autogrid support, the grids were first coated with an organometallic platinum layer using a gas injection system. Subsequently, the cells were milled with a gallium ion beam to produce ∼100- to 200-nm-thick lamellas, exposing the cellular interior.

### Cryo-ET.

EM grids containing lamellas were transferred into a 300-kV Titan Krios microscope (FEI Thermo Fisher), equipped with a postcolumn energy filter (Gatan) and a K2 Summit direct detector camera (Gatan). Using SerialEM software (76), tilt series were acquired with 2° steps between −60° and +60° (in two halves, separated at −0° or −20°). Individual tilts were recorded in movie mode at 12 frames per second, at an object pixel size of 3.42 Å and a defocus of −4 to −5.5 µm. The total accumulated dose for the tilt series was kept below ∼100 e^−^/Å^2^. Each tomogram was acquired from a separate cell and thus is both a biological and technical replicate. Several different cell cultures and >10 imaging sessions were used to produce the dataset.

### Tomogram Reconstruction.

Prior to reconstruction, the raw frames from the K2 detector were drift corrected with MotionCor2 software (77) using 3 × 3 patches, and then tilt-series stacks were assembled. Using IMOD software (78), the tilt-series were aligned with patch tracking and reconstructed with weighted backprojection to generate tomographic volumes. We used stringent quality control criteria including tilt-series alignment scores and the power spectra of individual tilts to, first, omit poor tilts from tomograms and, second, remove full tomograms from the dataset. Proteasomes, Cdc48, and ribosomes were extracted from 76, 14, and 6 tomograms, respectively. Contrast enhancement for display (Fig. 2) was performed with the tom_deconv deconvolution filter (https://github.com/dtegunov/tom_deconv).

### Tomogram Visualization and Segmentation.

Slices through tomogram volumes (Fig. 2 *A*–*C*) were generated with the IMOD 3dmod viewer. Tomogram segmentation (Fig. 2*D*) was performed in Amira software (FEI Thermo Fisher), with the help of automated membrane detection from the TomoSegMemTV package (79). The 3D cellular volumes with mapped-in complexes (Figs. 2*D*, 4 *A* and *B*, and 5*C* and *SI Appendix*, Figs. S6 and S7) were displayed in University of California, San Francisco (UCSF) Chimera (80).

### Template Matching.

Proteasomes, Cdc48, and ribosomes were template matched (81) in twice-binned tomograms (13.68-Å pixel size) by searching with down-filtered structures using PyTom software (82). Cytosolic proteasomes were used from the dataset acquired in ref. 5. All templates were based on single-particle cryo-EM structures (Electron Microscopy Data Bank: EMD-2594, EMD-3326, and EMD-2858) (30, 83, 84), down-filtered to 40 Å. The proteasome template was constructed by masking one cap from the double-capped proteasome structure, yielding accurate hits for both single- and double-capped proteasomes. The ribosome template was constructed by masking the ribosome structure’s small subunit, enabling the subsequent sorting of true hits from false positives. Following template matching, peak extraction by cross-correlation delivered a first set of possible candidates, which were screened with either classification in the case of ribosomes, or visual inspection for proteasomes and Cdc48. Ribosomes were classified by constrained PCA (CPCA) (85) for recovery of the small subunit that was omitted from the template, enabling us to discard noise and false picks.

### Subtomogram Averaging and Classification.

After the presorting described above, initial alignment of unbinned subtomograms (3.42-Å pixel size) was performed in PyTom software, using global alignment with spherical harmonics (86) to get a first estimate of the angles and shifts for proteasomes, Cdc48, and ribosomes. Proteasomes and Cdc48 were further refined with several rounds of real-space alignment and classification in PyTom and RELION (87), as described in ref. 5 for proteasomes and below for Cdc48. Refinement in RELION included internal normalization and contrast transfer function (CTF) correction with CTFFIND4 (88). PyTom subtomogram averages were instead corrected for CTF in IMOD. Resolution for proteasome and Cdc48 averages was determined in RELION by gold-standard Fourier shell correlation, whereas the ribosome resolution was estimated by cross-correlation with the highly resolved single-particle structure that was used for template matching (EMD-2858) (84) (*SI Appendix*, Fig. S9 *B*–*D*).

Several rounds of classification were performed to improve structural homogeneity and distinguish between various conformational states. Membrane-bound ribosomes were selected from the total cytosolic population with a distance restraint from the ER and nuclear membranes and subsequent classification of subvolumes with CPCA, as described in ref. 37. Using PyTom, proteasomes were sorted into single- or double-capped structures and ground- or processing-state caps, as described in ref. 5. Cdc48 complexes from all tomogram were classified in RELION using 6-fold symmetry. Several rounds of iterative classification using 2 to 3 classes resulted in a well-resolved “high-quality” class that resembled the single-particle cryo-EM structure (*SI Appendix*, Fig. S10). High-quality Cdc48 particles from the tomograms without proteasome clusters were combined with all Cdc48 candidates from the cluster tomograms for another round of classification; increasing number of well-resolved particles in the classification enabled reliable determination of Cdc48 within the cluster tomograms. The identity of Cdc48 was further confirmed by calculating a reference-free average, starting from a sphere of randomly aligned particles. This alignment was successfully performed both unsymmetrized (*SI Appendix*, Fig. S11) as well as with 6-fold symmetry (*SI Appendix*, Fig. S9). Although the unsymmetrized average resembled a mix of ring and staircase confirmations (*SI Appendix*, Fig. S12), no further reliable classification of Cdc48 was possible due to the small size of the complex (540 kDa).

### Fitting Molecular Models into EM Densities.

In order to visualize the extra density bound to the ER-associated proteasomes in Fig. 5*B*, a molecular model of the S1 ground-state proteasome (83) was fit into the EM density of the respective subtomogram average by rigid-body fitting in UCSF Chimera (89). Molecular models of the yeast 26S proteasome (PDB-5MP9) (90), human Cdc48/p97 (PDB-5FTK) (31), and yeast 80S ribosome (PDB-3J78) (91) determined by single-particle cryo-EM were also fit into their respective subtomogram averages using rigid-body fitting in UCSF Chimera (*SI Appendix*, Fig. S9 *B*–*D*).

### Clustering Behavior of Proteasomes and Cdc48.

We calculated pairwise distances between all of the cytosolic proteasomes in the tomograms. We then randomly placed the same number of proteasomes into the same cytosolic volumes (masked in Amira software to exclude the volumes of vesicles and organelles) while avoiding overlap, and again calculated the pairwise distances. The real data showed a clear peak at short distances that was absent from the random simulated data, revealing strong clustering behavior (*SI Appendix*, Fig. S8*A*). To define which proteasomes are within ER-associated clusters, the cytosolic proteasomes were divided into a varying number of groups using the *k*-means algorithm, and the quality of the fit was evaluated. Based on the local maximum of this distribution, we defined the proteasome population belonging to each cluster.

To determine whether Cdc48 clusters along with the proteasomes, we calculated the distances between every proteasome to every cytosolic Cdc48 (Fig. 4*E*). This calculation was performed independently for cluster and noncluster proteasomes, measuring to both the experimentally determined Cdc48 positions and also simulated data where Cdc48 complexes were randomly placed throughout the same cytosolic volumes (again, excluding vesicles and organelles). Whereas there was no significant difference between the real and random Cdc48 positions for noncluster proteasomes, the cluster proteasomes had a strong peak at <200 nm when measuring to the real Cdc48 positions. Thus, cytosolic Cdc48 has a nonrandom distribution that correlates with the positions of the clustered proteasomes. The statistical significance of this coclustering was confirmed by calculating the nearest-neighbor cumulative distribution, showing the distance to the closest Cdc48 for each cluster and noncluster proteasome (*SI Appendix*, Fig. S8*B*). Comparison of the distributions to a random simulation with a 2-sample Kolmogorov–Smirnov test supports highly statistically significant clustering. As active proteasomes and Cdc48 cluster together at the ER membrane, we have defined these distinct cytosolic regions as degradation microcompartments. For the Cdc48 complexes outside of the microcompartments, the distances between Cdc48 and the ER membrane were indistinguishable from a random simulation (*SI Appendix*, Fig. S8*D*).

As a control, the distances were calculated between every proteasome (cluster and noncluster) and every cytosolic ribosome (*SI Appendix*, Fig. S8*E*). The distributions were not significantly different for the cluster and noncluster proteasomes; thus, ribosomes do not cluster with proteasomes at the ER.

### Analysis of Degradation Microcompartment Shape and Composition.

Proteasome cluster shapes were evaluated by PCA to determine the eigenvalues for the first 3 principal components. The averages of the eigenvalues for all clusters were compared to eigenvalues of model shapes (2D circle and 3D sphere, Fig. 4*C*).

To dissect the molecular architecture of the degradation microcompartments, we calculated the concentrations of proteasomes, Cdc48, and ribosomes in evenly spaced shells expanding from the centroids of each cluster (Fig. 4*D*). The centroid was calculated by averaging all cluster proteasome positions, while the concentration was determined by counting the number of particles within the shell and dividing it by the shell’s volume. The shell’s volume included only the cytosol, excluding the volumes of organelles such as the ER, Golgi, and mitochondria.

### Statistical Analysis of Proteasome Functional States within Degradation Microcompartments.

To test whether proteasomes within 20 nm of the ER membrane (14 caps, 50% processing state) were significantly more active than proteasomes in the rest of the ER-associated cluster (159 caps, 17% processing state), we calculated the probability of randomly drawing 14 caps that consisted of least 50% processing state from the pool of clustered proteasome caps further than 20 nm from the ER. To simplify the calculation, 16 unclassifiable proteasome caps (9% of the total population) were neglected. A numeric calculation of probabilities with and without the unclassifiable caps showed that this simplification is justified (*SI Appendix*, Fig. S16). According to the urn model without replacement and order, the probability was calculated as follows:

$$\sum_{k1=7:14,k2=7:1}P\left(X_{1}=k_{1},X_{2}=k_{2}\right)=\sum_{k1=7:14,k2=7:1}\frac{\left(\begin{array}{c} N_{1}\\ k_{1} \end{array}\right)\left(\begin{array}{c} N_{2}\\ k_{2} \end{array}\right)}{\left(\begin{array}{c} N\\ n \end{array}\right)},$$

with *N*_1_ = 29 (number of processing states > 20 nm); *N*_2_ = 130 (number of ground states > 20 nm); *N* = 159 (number of caps > 20 nm); *n* = 14 (number of caps drawn); *k*_1_ = 7:14 (number of drawn processing states); *k*_2_ = 1:7 (number of drawn ground states); *k*_1_ + *k*_2_ = *n* = 14.

The probability of drawing 14 caps with at least 50% processing state is only 0.3%. Therefore, we can exclude with high significance that the increased percentage of processing states for proteasomes <20 nm from the ER membrane is random.

### Whole-Cell Mass Spectrometry.

*Chlamydomonas mat3-4* cells were grown to midlog phase in the same conditions as for the cryo-ET, pelleted at 1,000 × *g* for 5 min, and flash-frozen in liquid nitrogen. The cell pellet was lysed in 3 mL of buffer containing 1% sodium deoxycholate (SDC), 20 mM TCEP, and 40 mM chloroacetamide in 25 mM Tris, pH 8.5. The lysate was heated to 95 °C for 2 min, followed by sonication in a Bioruptor sonicator (Diagnode) at maximum power (10 cycles of 30-s pulses). The heating and sonication was repeated once to ensure complete lysis. One hundred microliters of lysate were mixed with 100 µL of LC-MS grade water, vortexed briefly, and incubated at 37 °C for 30 min. After reduction and alkylation, the samples were digested overnight with 4 µg of trypsin. Following overnight digestion, the sample was acidified in a final concentration of 1% trifluoroacetic acid to precipitate the SDC. The supernatant was then used for purification and fractionation in a SCX StageTip (92). All three fractions from the purification were loaded into a 15-cm-long and 75-µm-wide column (New Objective), packed with 1.9-µm C18 reprosil beads (Dr Maisch GmbH). The peptides were eluted over a gradient for ∼140 min and directly sprayed into a bench top orbitrap mass spectrometer (Q Exactive HF; Thermo Fisher) (93). Data-dependent acquisition was employed, and the top 15 precursors were subjected to HCD-based fragmentation (94). The raw data were processed using the MaxQuant computational platform, version 1.6.0.15 (95), and searched against the *Chlamydomonas* proteome derived from all 19,526 protein-coding transcripts of the *Chlamydomonas* genome, version 5.5 (96, 97), using the Andromeda search engine (98) with initial parent ion mass deviation of 6 ppm. All identifications were filtered at a 1% false-discovery rate. As a proxy for protein abundance, intensity-based absolute quantification (iBAQ) values (99) were generated and used to compare the different protein complexes within the sample (Fig. 3).

### Bioinformatic Identification of Conserved ERAD Components in *Chlamydomonas reinhardtii* and *Arabidopsis thaliana*.

*Chlamydomonas* homologs to the core ERAD components (*SI Appendix*, Table S1) present in version 5.5 of the *Chlamydomonas* genome (96, 97) were identified by BLAST searches using the Phytozome platform (100) and the BLAST platform at National Center for Biotechnology Information (NCBI) (https://blast.ncbi.nlm.nih.gov/Blast.cgi) using *Saccharomyces cerevisiae*, *Homo sapiens*, and *Arabidopsis thaliana* protein sequences as queries. All homologs were confirmed by manual sequence alignment to confirm the conservation of functional protein domains. Domain composition was determined with the Simple Modular Architecture Research Tool (http://smart.embl-heidelberg.de). For Cue1-like proteins, the full SMART annotation of the Cue domain included a list of all proteins containing a Cue domain. Only one *Arabidopsis* protein contained a single Cue domain with no additional functional domains; this sequence was used as a query to identify the matching *Chlamydomonas* protein. A BLAST search using the yeast Cue1 and human CueD1 proteins failed to identify a similar protein in *Chlamydomonas* or *Arabidopsis*.

### Seed Extension by GRAbB.

To resolve some of the gene models encoding proteins with large low-complexity stretches, we performed a local, seeded de novo assembly from RNA-sequencing (RNA-seq) reads with the program GRAbB (101). RNA-seq reads came from run SRR2132411 from the Short Read Archive SRA at NCBI, from a diurnal time course, 10 h into the light part of the day (102). Briefly, we chose as seed a sequence with good read coverage that did not overlap with any dubious part of the gene model. GRAbB identified all reads mapping to the seed and assembled them into a new seed for the next iterative round.

For NPL4 (Cre06.g293051), the current gene model encodes a protein with similarity to an ammonium transporter at its N terminus, and a protein with 2 NPL4 domains at its C terminus. However, the gene model tries to accommodate a gap in sequence of about 5,000 bp, in addition to low-complexity sequences on either side of the gap. We used 918 bp downstream of the gap as the seed. After 5 rounds of extension, GRAbB assembled reads into a final 1,683 bp in silico cDNA encoding a protein of 426 amino acids with 3 Npl44-like domains (*SI Appendix*, Fig. S2).

For HRD1 (Cre04.g217922), we used a 729-bp seed that mapped to the penultimate exon, upstream of a transposon-like element in the last intron. After 8 rounds, GRAbB assembled reads into a 1,121-bp in silico cDNA, encoding a 333-amino acid protein that includes the 6 transmembrane domains and the RING domain. The position of the STOP codon is supported by PASA-assembled expressed sequence tags (103); the gene model should therefore not include the 3′ end encoding the C-terminal low-complexity region.

For Cue1 (Cre13.g568750), we used a 128-bp seed corresponding to the Cue domain. After 5 rounds, GRAbB assembled reads into a 543-bp in silico cDNA, encoding a 181-amino acid protein that lacks most of the low-complexity regions found in the current gene model.

For EDEM1 (Cre06.g301600), we used a 2,052-bp seed that covers the entire mannosidase-encoding portion of the gene. After 5 rounds, GRAbB assembled reads into a 2,320-bp in silico cDNA, encoding a 732-amino acid protein consisting of a signal peptide at its N terminus, the mannosidase domain (used as seed), and a small low-complexity region at its C terminus.

### Data Availability:

Subtomogram averages and an example tomogram have been deposited in the Electron Microscopy Data Bank (EMD-3932 to EMD-3935 and EMD-10409 to EMD-10411). Mass spectrometry data have been deposited in the PRIDE archive (PXD009375).

## Supplementary Material

Supplementary File

Supplementary File

Supplementary File

Supplementary File

Supplementary File
