# Supplementary material for: Direct visualization of degradation microcompartments at the ER membrane
Source: Proc Natl Acad Sci U S A. 2019 Dec 27;117(2):1069–80. doi: 10.1073/pnas.1905641117 (PMC6969544; doi:10.1073/pnas.1905641117)
Supplement: Supplementary File [file pnas.1905641117.sapp.pdf]

# Supporting Information (SI Appendix) for:
# Direct visualization of degradation microcompartments at the ER membrane

**Authors:** Sahradha Albert[1], Wojciech Wietrzynski[1#], Chia-Wei Lee[1#], Miroslava Schaffer[1#], Florian Beck[1], Jan M. Schuller[2], Patrice A. Salomé[3], Jürgen M. Plitzko[1], Wolfgang Baumeister[1]*, Benjamin D. Engel[1†]*

**Affiliations:**

[1]Department of Molecular Structural Biology, Max-Planck-Institute of Biochemistry, 82152 Martinsried, Germany.

[2]Department of Structural Cell Biology, Max-Planck-Institute of Biochemistry, 82152 Martinsried, Germany.

[3]Department of Chemistry and Biochemistry, UCLA, 951569, 607 Charles E. Young Drive East, Los Angeles, California, USA.

[†]Current address: Helmholtz Pioneer Campus, Helmholtz Zentrum München, Ingolstädter Landstraße 1, 85764 Neuherberg, Germany.

# Equal contribution

* Corresponding authors:

Wolfgang Baumeister

Department of Molecular Structural Biology

Max Planck Institute of Biochemistry

82152 Martinsried, Germany

+49-(0)89-8578-2592

baumeist@biochem.mpg.de

Benjamin Engel

Department of Molecular Structural Biology

Max Planck Institute of Biochemistry

82152 Martinsried, Germany

+49-(0)89-8578-2653

engelben@biochem.mpg.de

www.pnas.org/cgi/doi/10.1073/pnas.1905641117

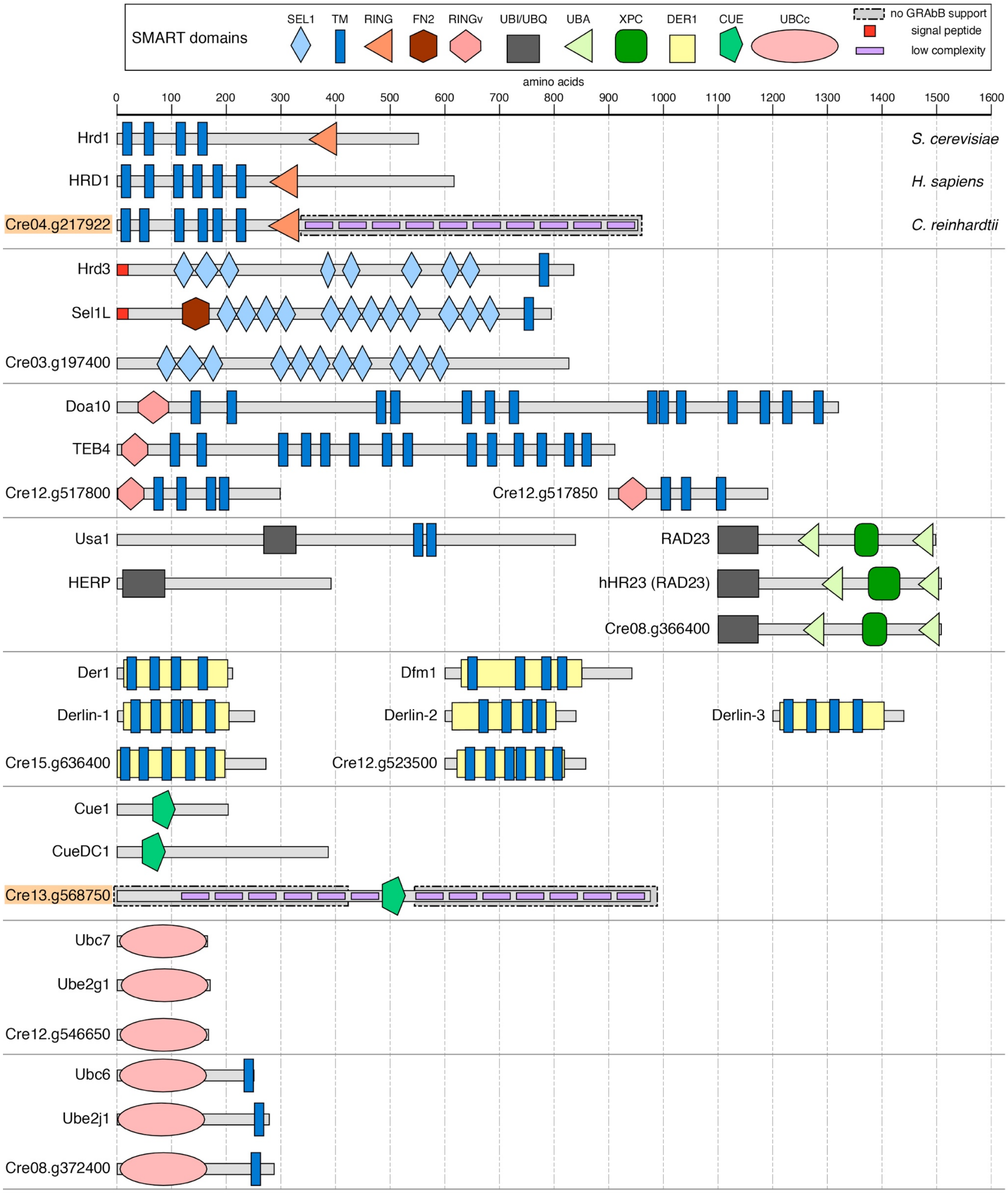


**Figure S1. Functional domain organization of ERAD components in *Saccharomyces cerevisiae*, *Homo sapiens*, and *Chlamydomonas reinhardtii*.** All protein sequences were retrieved from NBCI (for yeast and human proteins) or Phytozome (1) (for *Chlamydomonas*) and submitted to SMART (Simple Modular Architecture Research Tool, http://smart.embl-heidelberg.de) for domain detection. *Chlamydomonas* proteins with gene names highlighted in color have large stretches of low complexity, which are not supported by GRAbB-generated in silico cDNAs (SI Appendix, Fig. S2) and may denote an incorrect gene model. All orthologous proteins are shown in the order: yeast, human, *Chlamydomonas*.

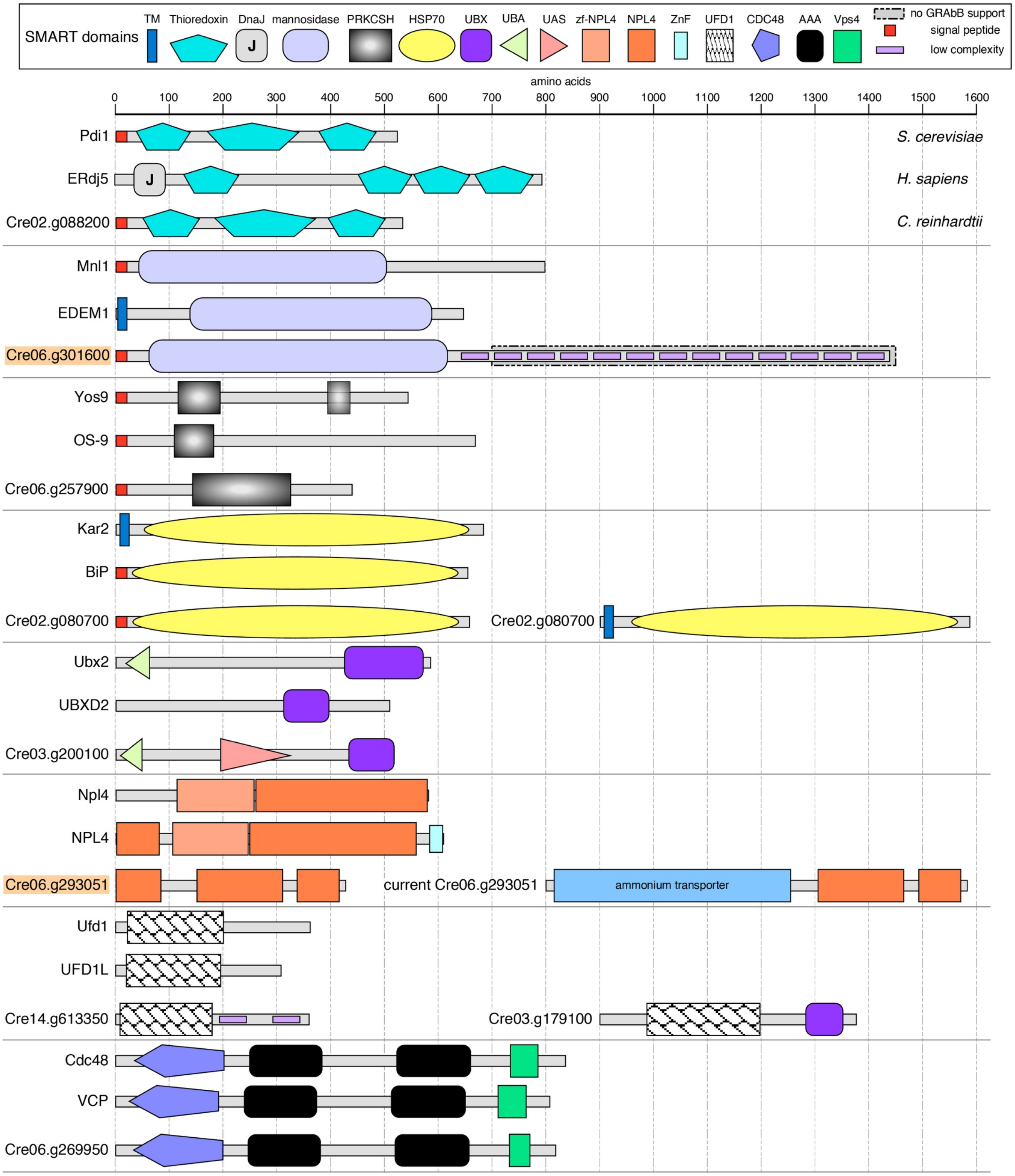


**Figure S1 (continued). Functional domain organization of ERAD components in *Saccharomyces cerevisiae*, *Homo sapiens*, and *Chlamydomonas reinhardtii*.** All protein sequences were retrieved from NBCI (for yeast and human proteins) or Phytozome (1) (for *Chlamydomonas*) and submitted to SMART (Simple Modular Architecture Research Tool, http://smart.embl-heidelberg.de), for domain detection. *Chlamydomonas* proteins with gene names highlighted in color have large stretches of low complexity, which are not supported by GRAbB-generated in silico cDNAs (SI Appendix, Fig. S2) and may denote an incorrect gene model. All orthologous proteins are shown in the order: yeast, human, *Chlamydomonas*.

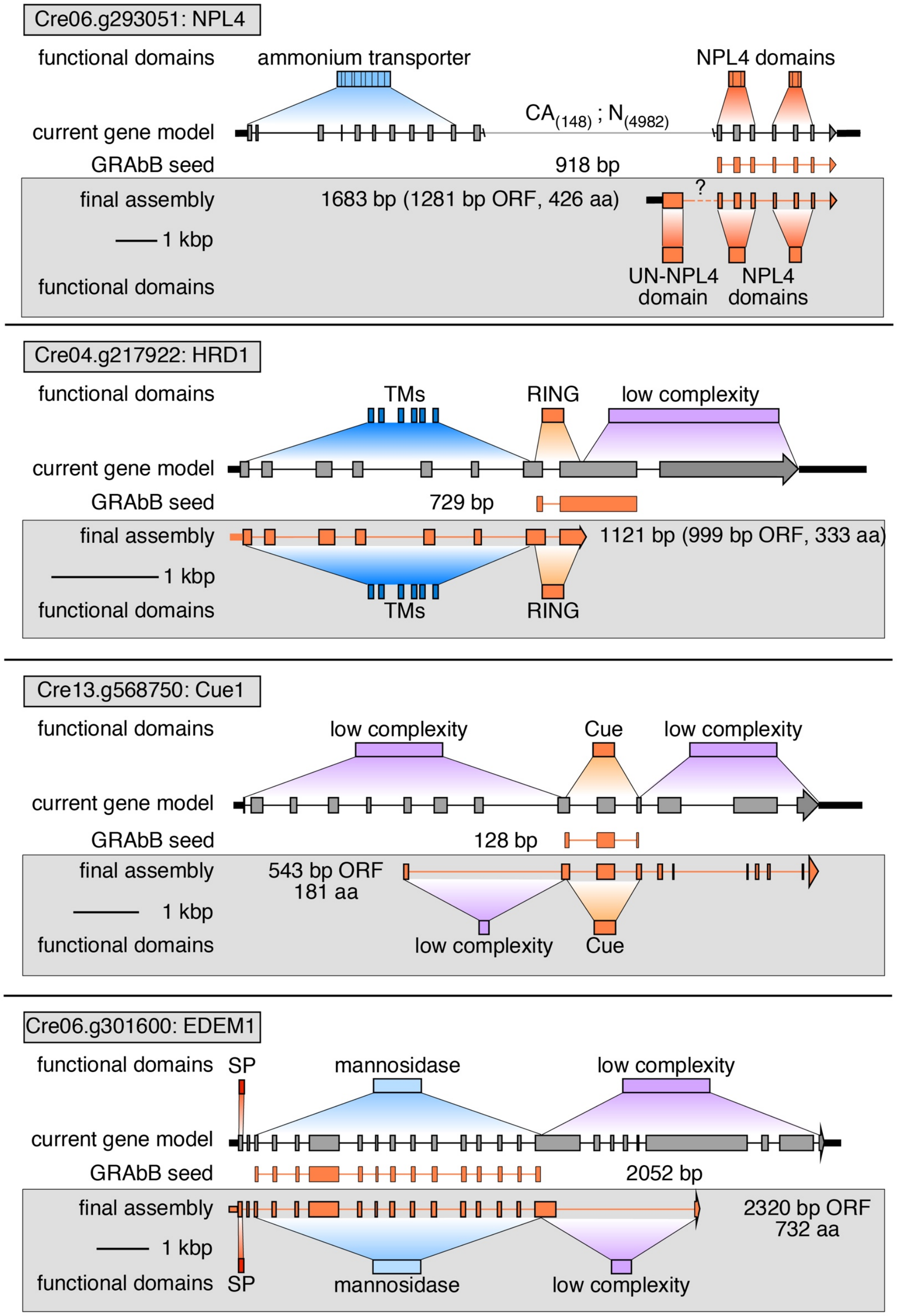


**Figure S2. Resolving incorrect gene models for the *Chlamydomonas* NPL4, HRD1, Cue1 and EDEM1 orthologs.** We used GRAbB (2) to assemble the full coding sequences corresponding to the C*hlamydomonas* NPL4, HRD1, Cue1 and EDEM1 genes. The sequences were assembled from a paired-end RNAseq experiment with high sequence coverage (run SRR2132411 from the Short Read Archive SRA at NCBI), using only the parts of the genes that code for expected functional domains as seeds. The final GRAbB sequences and deduced protein sequences (with functional domains) are shown in the grey boxes below each GRAbB seed.

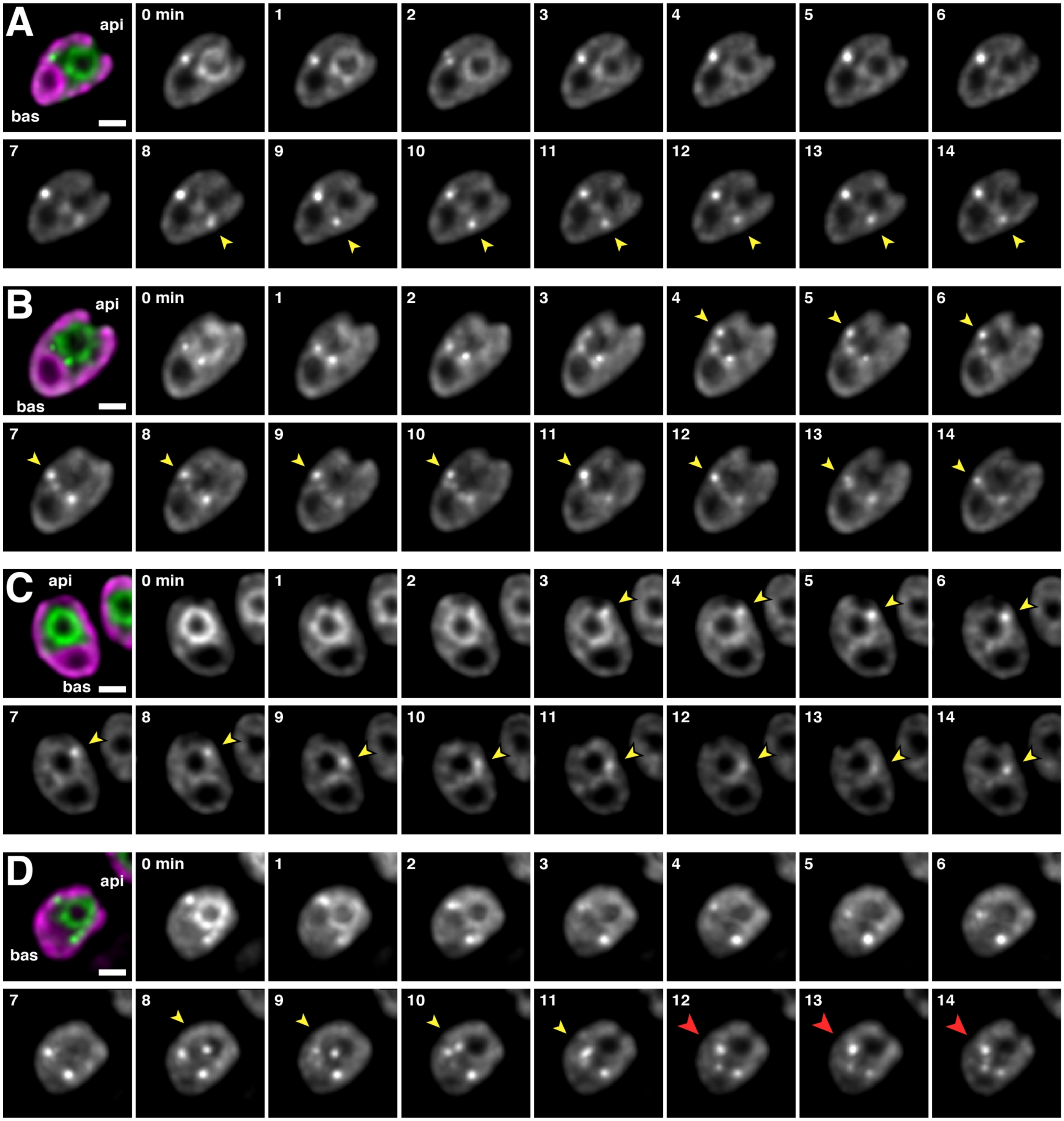


**Figure S3. Cytosolic proteasome puncta dynamically assemble. (A-D)** Time series of live *Chlamydomonas mat3-4* cells expressing Rpn11-mVenus. 3D Z-stacks were acquired once per minute by widefield deconvolution fluorescence microscopy. The first image of each time series is an overlay of Rpn11-mVenus (green) and chlorophyll autofluorescence (magenta) at 0 min, displaying the apical (api) and basal (bas) sides of the cell. The greyscale images in each time-series track Rpn11-mVenus over 14 minutes, showing maximum intensity projections through all Z-slices containing puncta**.** Small yellow arrowheads indicate newly assembled puncta, with clear signal that appears and can be tracked over time, despite significant photobleaching during the time series. The large red arrowhead in D is a fusion event between the newly assembled punctum and a preexisting punctum. See Movie 1.

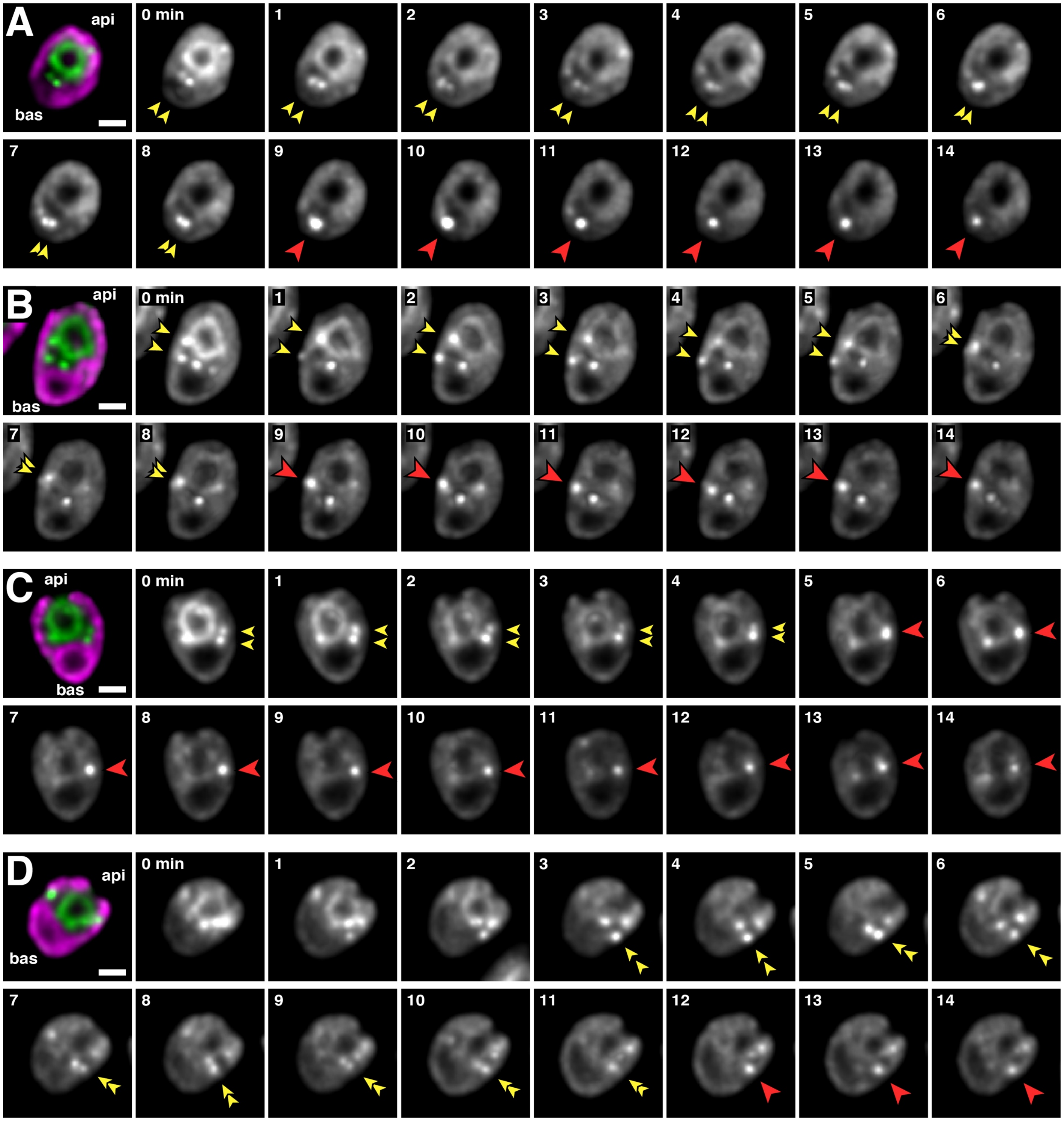


**Figure S4. Cytosolic proteasome puncta dynamically fuse. (A-D)** Time series of live *Chlamydomonas mat3-4* cells expressing Rpn11-mVenus. 3D Z-stacks were acquired once per minute by widefield deconvolution fluorescence microscopy. The first image of each time series is an overlay of Rpn11-mVenus (green) and chlorophyll autofluorescence (magenta) at 0 min, displaying the apical (api) and basal (bas) sides of the cell. The greyscale images in each time-series track Rpn11-mVenus over 14 minutes, showing maximum intensity projections through all Z-slices containing puncta**.** Small yellow arrowheads indicate two puncta before fusion. Large red arrowheads indicate puncta after fusion. Despite significant photobleaching during the time series, fused puncta often have increased intensity due to the accumulation of more fluorescent protein within a diffraction-limited spot. See Movie 2.

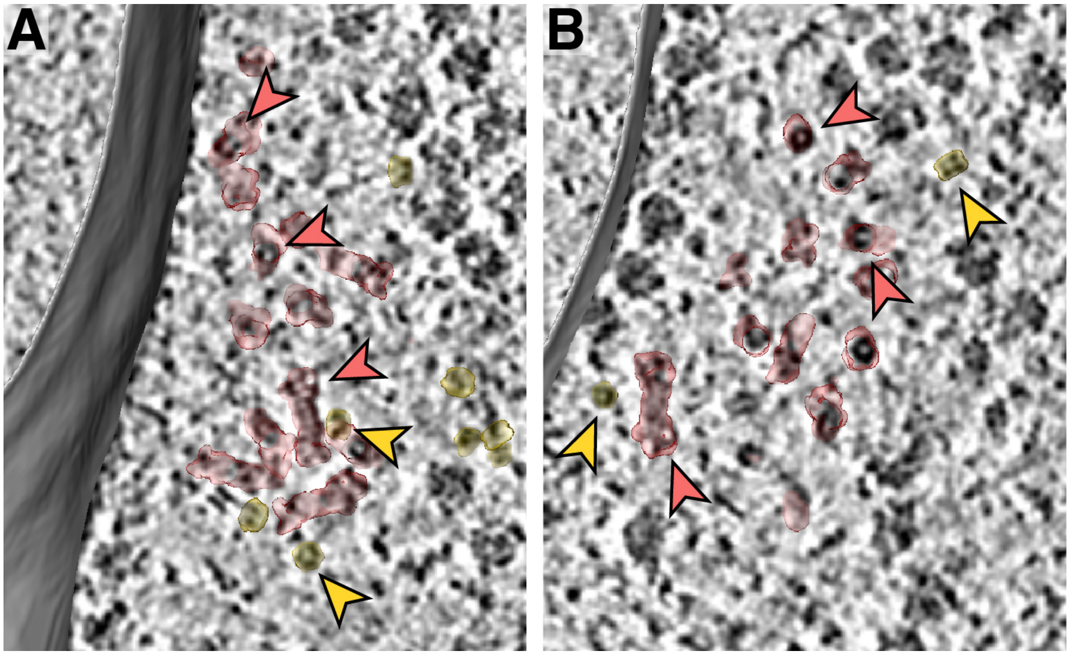


**Figure S5. Accurate identification of proteasomes and Cdc48 within a degradation microcompartment. (A-B)** Still frames from Movie 4 corresponding to the two Z-slices in Fig. 2B-C, with the same arrows pointing to proteasomes (red) and Cdc48 complexes (yellow). Transparent red and yellow silhouettes correspond to the mapped in subtomogram averages of proteasomes and Cdc48, respectively, as shown in Fig. 2D. A clipping plane has been used to only show the averages that are immediately above each tomogram Z-slice, allowing comparison between the placed 3D structures and the tomogram.

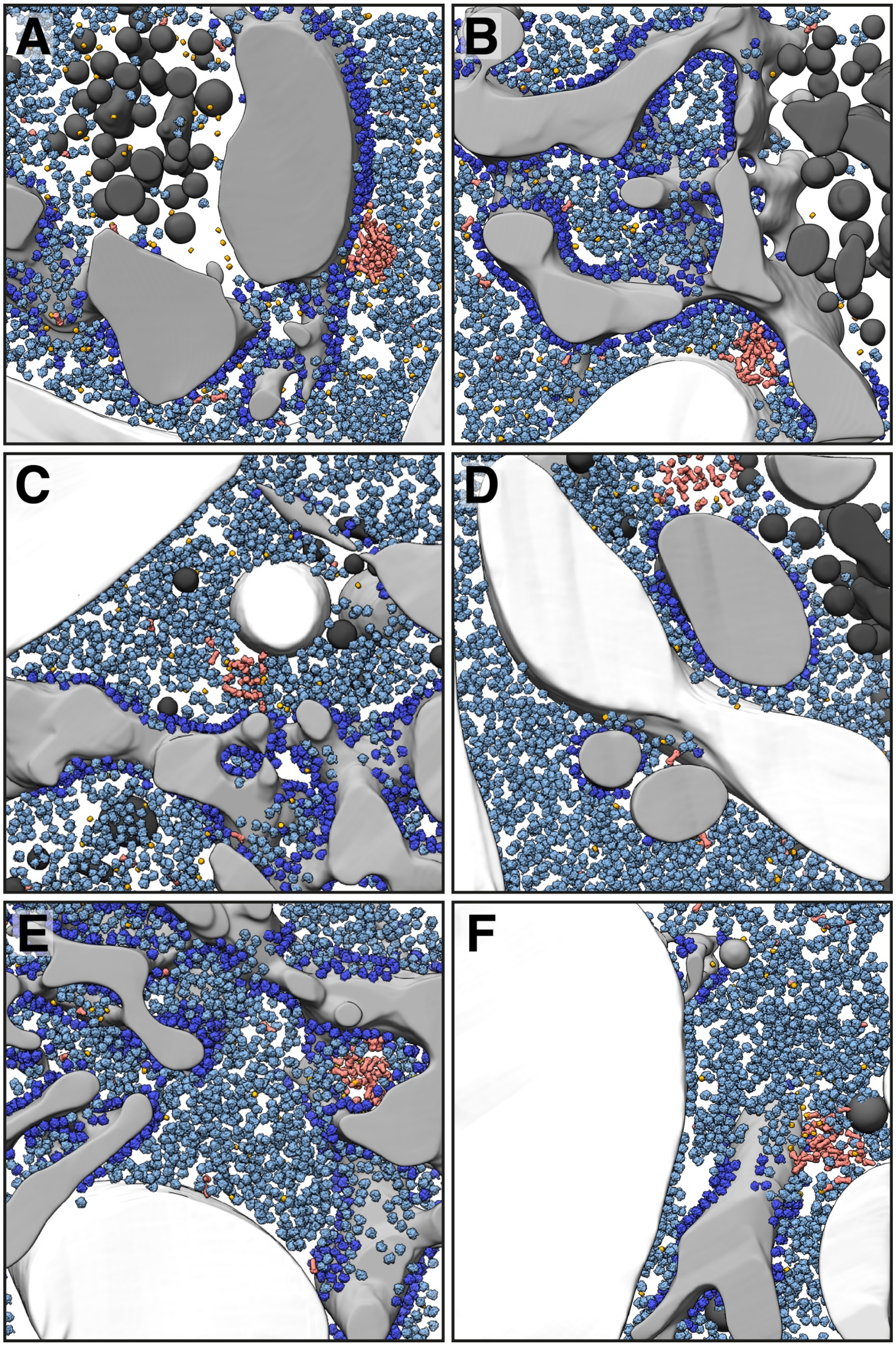


**Figure S6. Overviews of tomograms containing degradation microcompartments. (A-F)** Six different cells. The ER (light grey), Golgi (dark grey), and other organelles including mitochondria and the nucleus (white) were segmented and subtomogram averages of proteasomes (red), Cdc48 (yellow) and ribosomes (membrane-bound: dark blue, free: light blue) were mapped into the cellular volumes. Proteasomes and Cdc48 cluster together in microcompartments adjacent to the ER, in regions that are distinct from the ER exit sites where proteins are secreted to the Golgi.

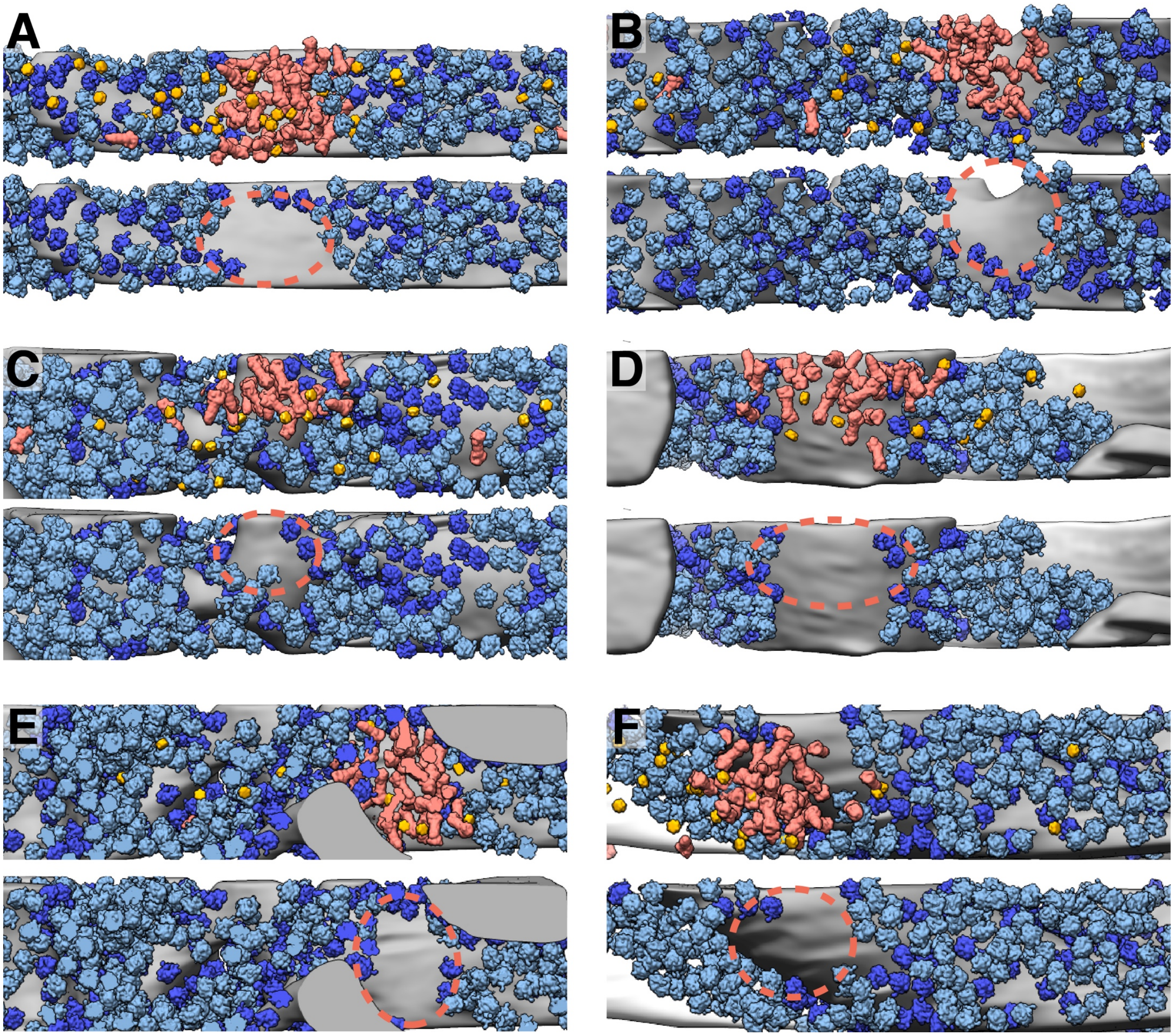


**Figure S7. Close-up views of degradation microcompartments at the ER membrane. (A-F)** The same six cells as in Fig. S6. Front views of the segmented ER membrane (grey) from the cytosol, showing examples of the degradation microcompartments. Proteasomes (red) and Cdc48 (yellow) cluster together at the membrane (top), whereas ribosomes (membrane-bound: dark blue, free: light blue) are excluded from the corresponding membrane and cytosolic region, marked with a red dashed line (bottom).

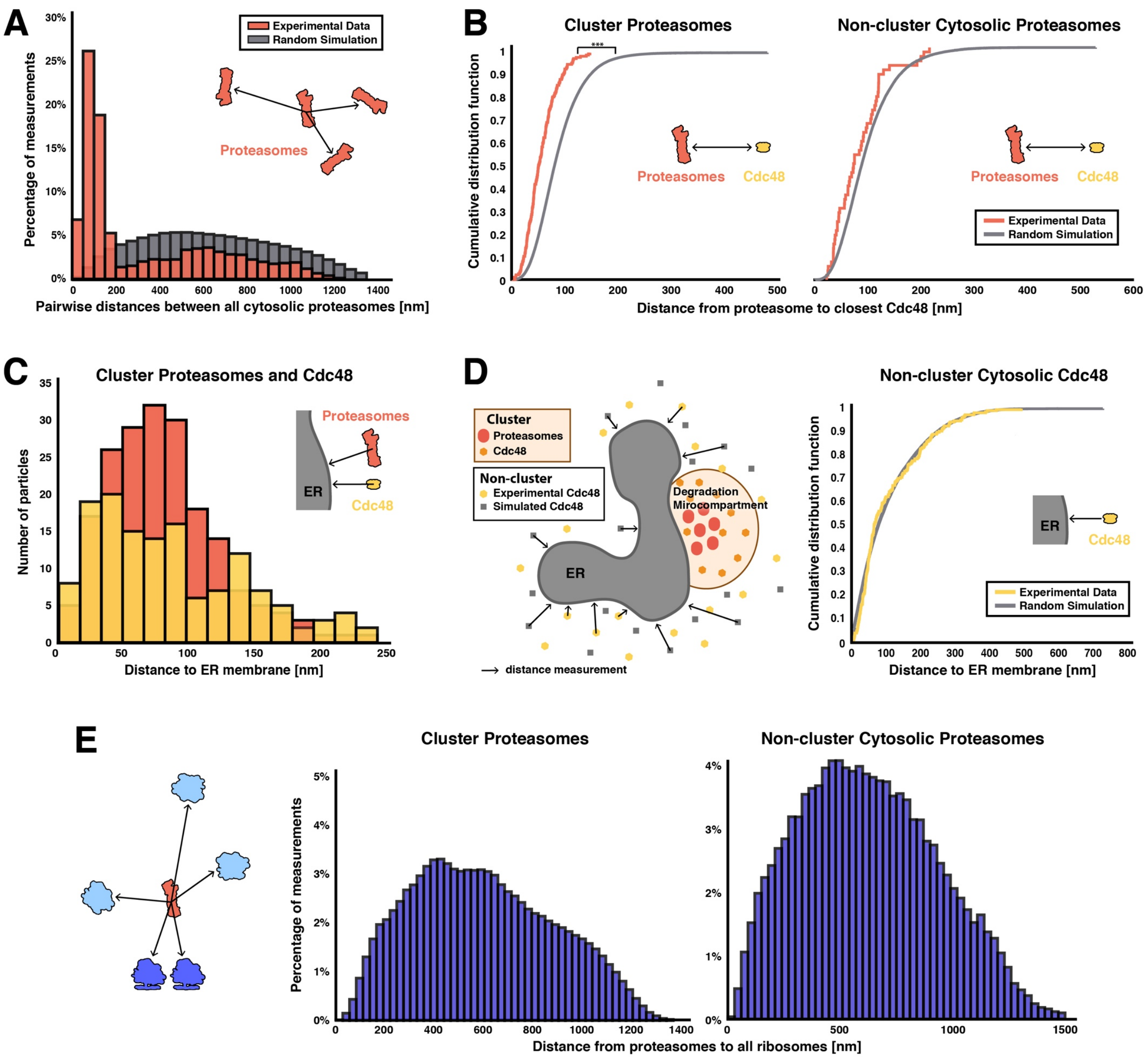


**Figure S8. Inter-particle distance analysis of proteasomes and Cdc48. (A)** Pairwise distance analysis between all cytosolic proteasomes (red), compared to randomly simulated proteasome positions (grey), showing a clear non-random peak at short distances that indicates clustering behavior. **(B)** Cumulative distributions of the distances between proteasomes and their nearest Cdc48 neighbor, for cluster proteasomes (left) and cytosolic non-cluster proteasomes (right). Comparing the distribution of measured Cdc48 positions (red) with randomly simulated Cdc48 positions (grey) reveals a highly significant non-random accumulation of Cdc48 at the cluster proteasomes. Kolmogorov-Smirnov test for cluster proteasomes: h=1 (different from random) with p=0.001 (***); for non-cluster proteasome: h=0 (same as random) with p=0.0849. **(C)** Distances of cluster-associated proteasomes (red) and Cdc48 (yellow) to the ER membrane, showing a similar distribution. **(D)** The cumulative distribution of distances between non-cluster Cdc48 and the ER membrane is indistinguishable from the distribution with randomly positioned Cdc48. Thus, while 80% of ER-proximal Cdc48 is found outside the degradation microcompartments, it appears to be randomly distributed along the ER membrane instead of clustered. **(E)** Distance analysis of each proteasome to all cytosolic ribosomes was performed for cluster proteasomes only (left) and for cytosolic non-cluster proteasomes (right), showing no correlation.

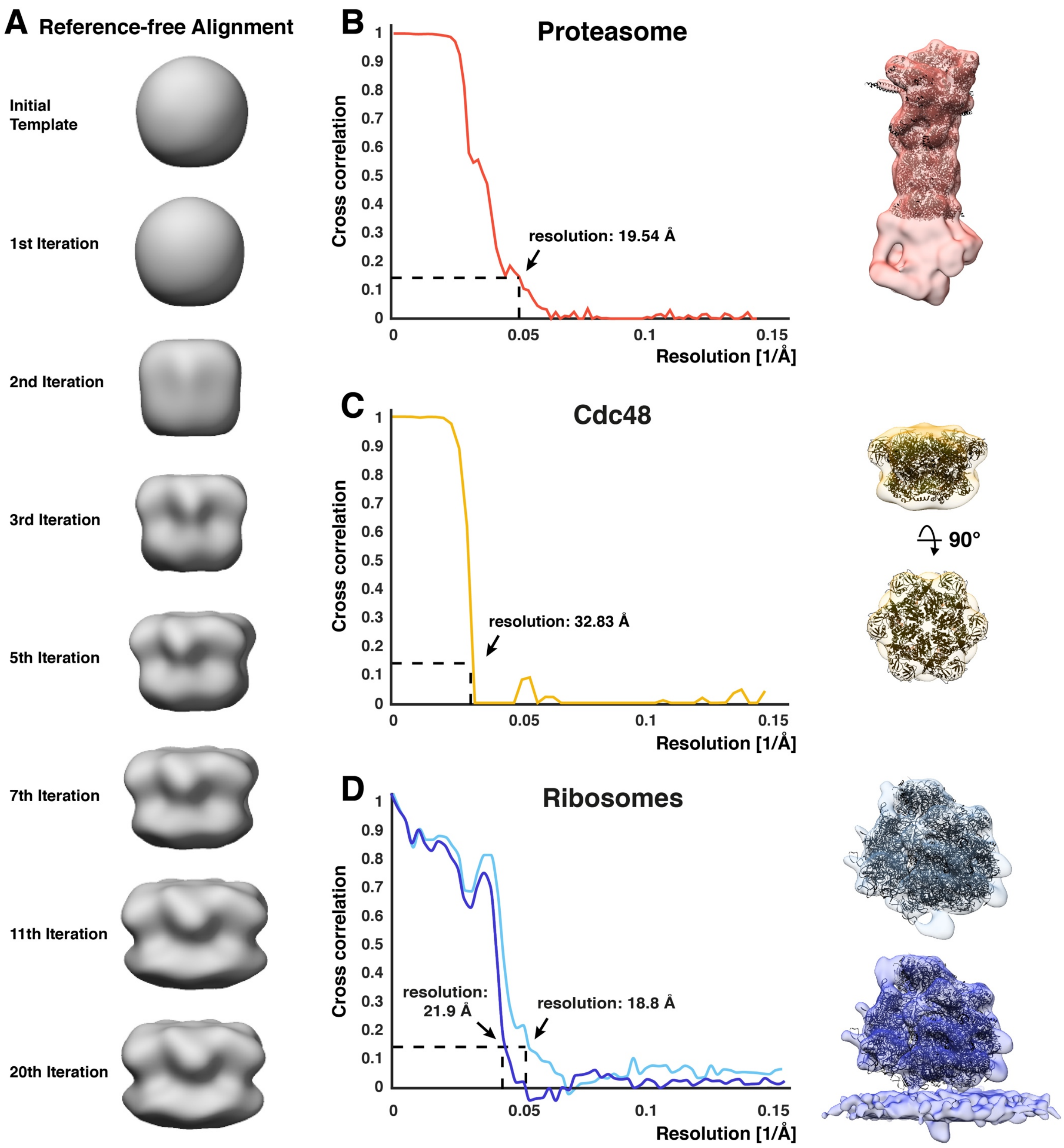


**Figure S9. Subtomogram averaging, resolution determination, and fitting. (A)** Reference-free subtomogram alignment of Cdc48 with random initial orientations. **(B-D)** Fourier shell correlation (FSC) curves (**left**) and rigid body fitting of molecular models (PDB-5MP9, PDB-5FTK, PDB-3J78) (3-5) (**right**) for the *in situ* subtomogram averages of **(B)** proteasomes, **(C)** Cdc48, and **(D)** cytosolic ribosomes (light blue: free, dark blue: membrane-bound). The proteasome model is single-capped. Fitted structures are not shown to scale with each other. The FSCs in **B** and **C** were calculated by gold-standard assessment in Relion and the FSC in **D** was instead calculated by a cross-correlation map with a highly-resolved single particle structure. Resolution was determined by the 0.143 cutoff criterion, drawn with a dashed line.

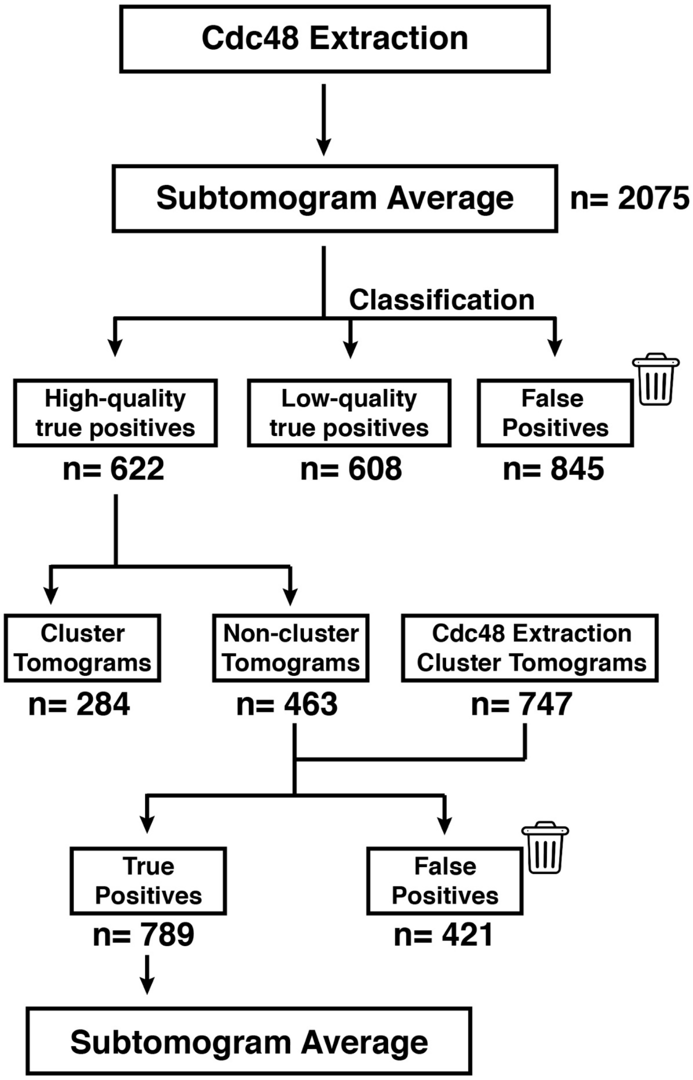


**Figure S10. Classification tree for generating the Cdc48 subtomogram average.** 2075 particles were extracted from 14 tomograms and averaged. Iterative classification separated 845 false positives to be discarded from highly-resolved and poorly-resolved classes of true positives. To enhance the identification of Cdc48 in the tomograms containing proteasome clusters (degradation microcompartments), all potential Cdc48 particles extracted from those cluster tomograms were classified together with the high-quality true positives from the non-cluster tomograms. The resulting 789 true positives Cdc48 complexes were averaged together for the final structure.

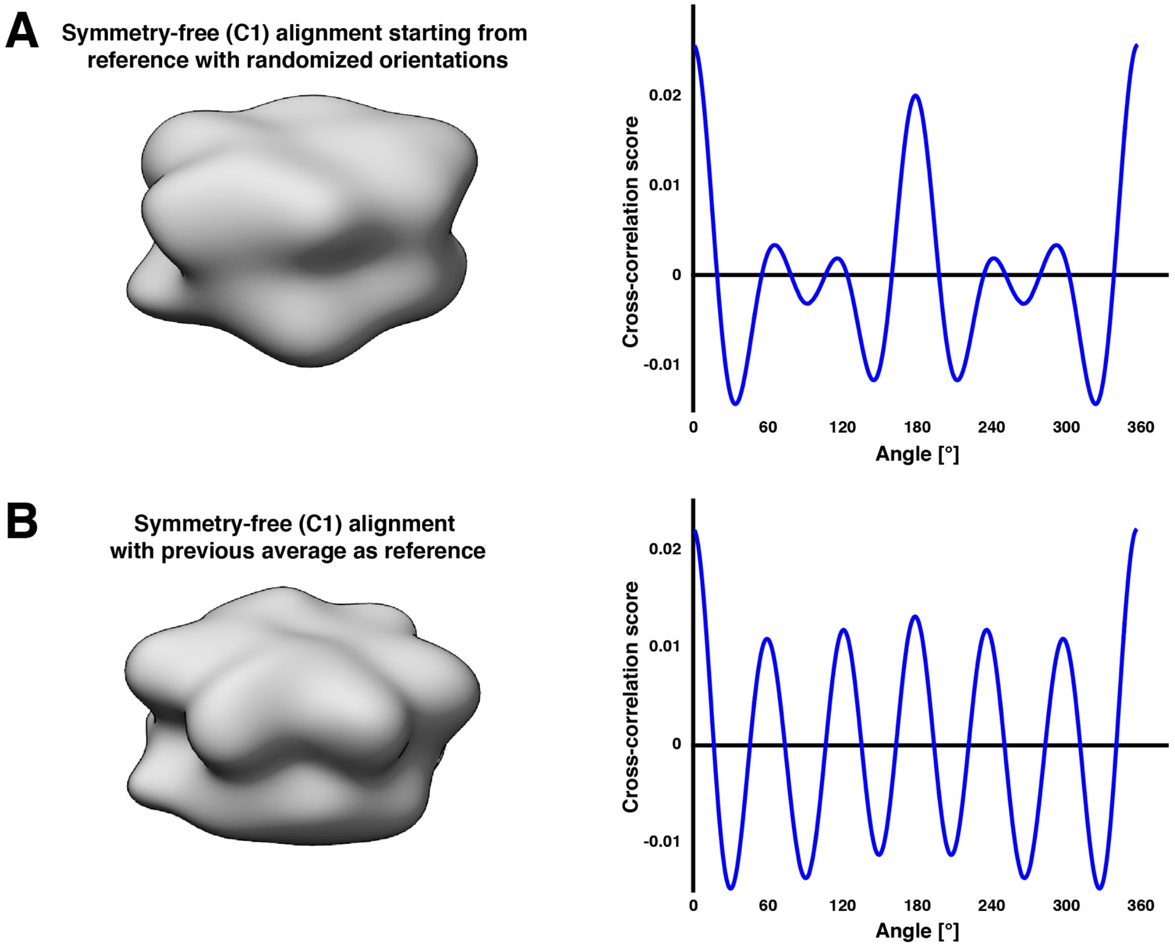


**Figure S11. Symmetry-free subtomogram averages of Cdc48.** Subtomogram average (left) and self-cross-correlation (autocorrelation) curve for different rotation angles, showing the 6-fold symmetry (right) for a non-symmetric reference-free alignment **(A)** and a non-symmetric reference-based alignment **(B)**, respectively.

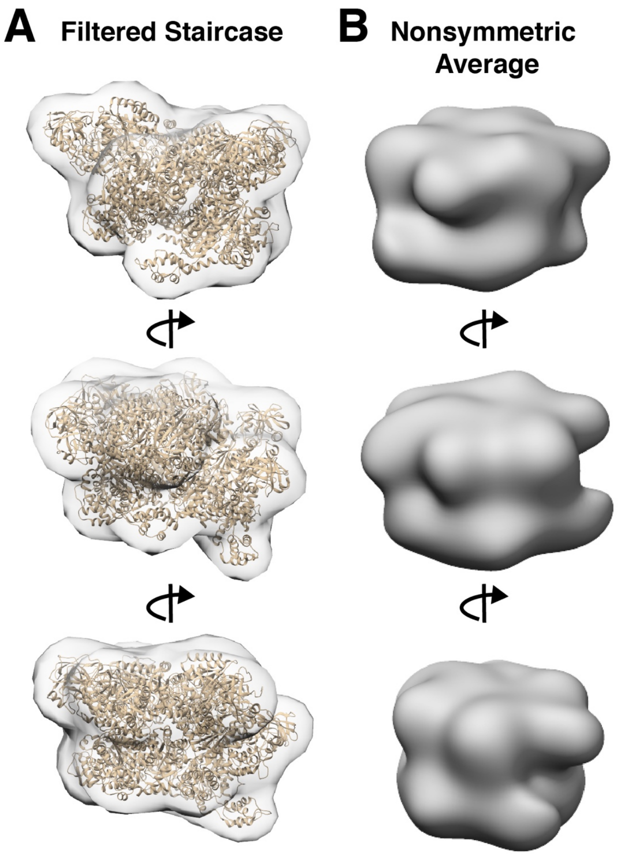


**Figure S12. The non-symmetrized Cdc48 average resembles a mix of ring and staircase conformations.** Comparison between **(A)** the Cdc48 single-particle cryo-EM structure in an "activated staircase" state (EMD-5G4F, brown: molecular model, transparent grey: calculated EM density) (6) and **(B)** the non-symmetrized reference-free Cdc48 average from our tomograms (SI Appendix, Fig. S11A). The asymmetric features of the two structures are similar, indicating that the *in situ* Cdc48 subtomogram average consists of a mix of ring and staircase particles. Thus, Cdc48 within the cellular tomograms is found in both ground and active conformational states.

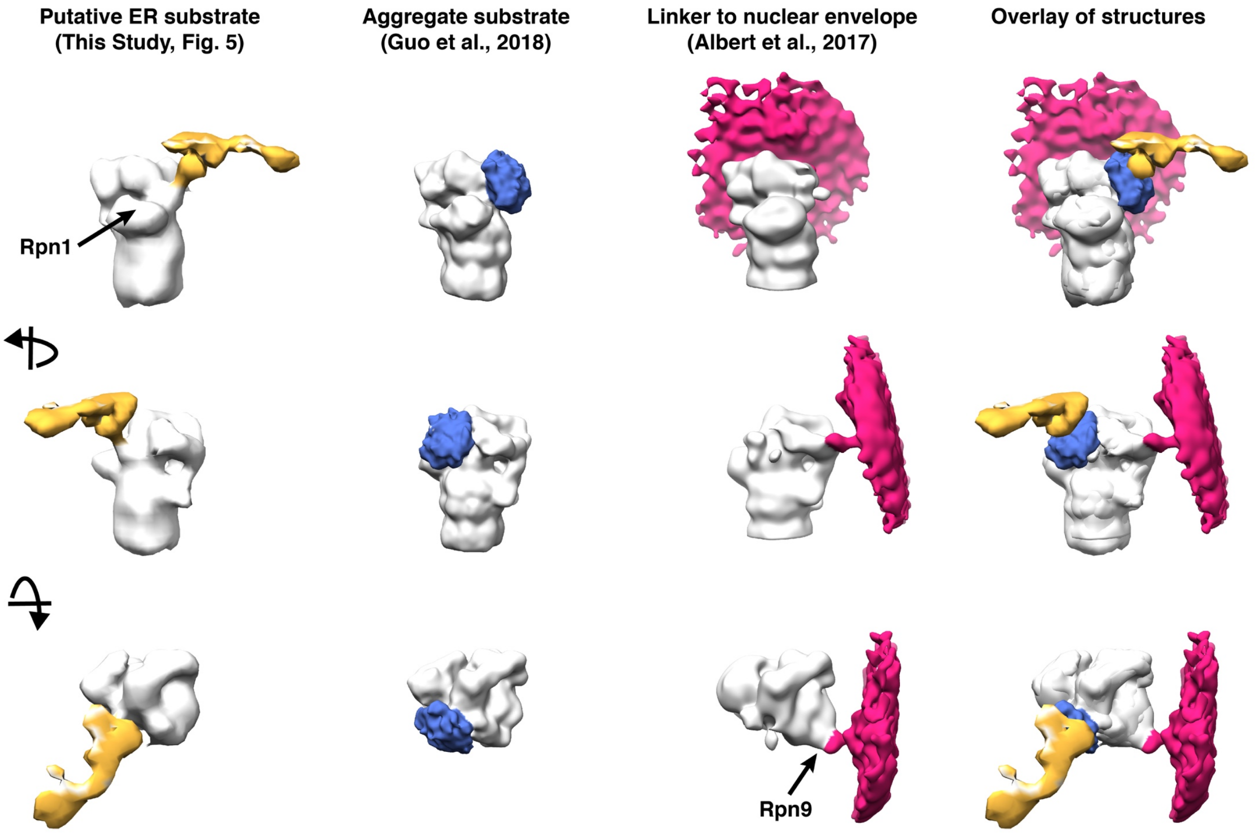


**Figure S13. Proteasomes at the ER membrane are bound to putative substrate.** Comparison of three *in situ* subtomogram averages of proteasomes (white) bound to extra densities (orange, blue, pink). The three rows show three views of the structures, as indicated by the rotation symbols. **Left column:** ER-proximal proteasomes within the degradation microcompartments, with 19S caps that are in the substrate-processing conformational state and are bound to an orange extra density that originates from the ER membrane (Fig. 5B-C). The extra density is bound to the proteasome's Rpt AAA-ATPase ring near the cap's Rpn1 subunit. **Middle-left column:** substrate-processing state proteasomes bound to poly-GA neurodegenerative aggregates, with the blue extra density corresponding to the engaged aggregate, a known substrate (EMD-3915) (7). This extra density is also bound to the AAA-ATPase ring near Rpn1. **Middle-right column:** proteasomes localized to the nuclear pore complex, with pink extra density corresponding to a linker protein and the inner nuclear membrane (EMD-3936) (8). The linker density binds the Rpn9 subunit of the proteasome's 19S cap. **Right column:** overlay of the three averages, fitted to each other in UCSF Chimera software (9). The density originating from the ER (orange) occupies the same binding position on the proteasome as the neurodegenerative aggregate substrate (blue). Thus, the orange density is very likely substrate that is engaged by the proteasome. As the orange density emanates from the ER membrane (Fig. 5C), we conclude that these proteasomes are likely engaged with ER-localized substrates.

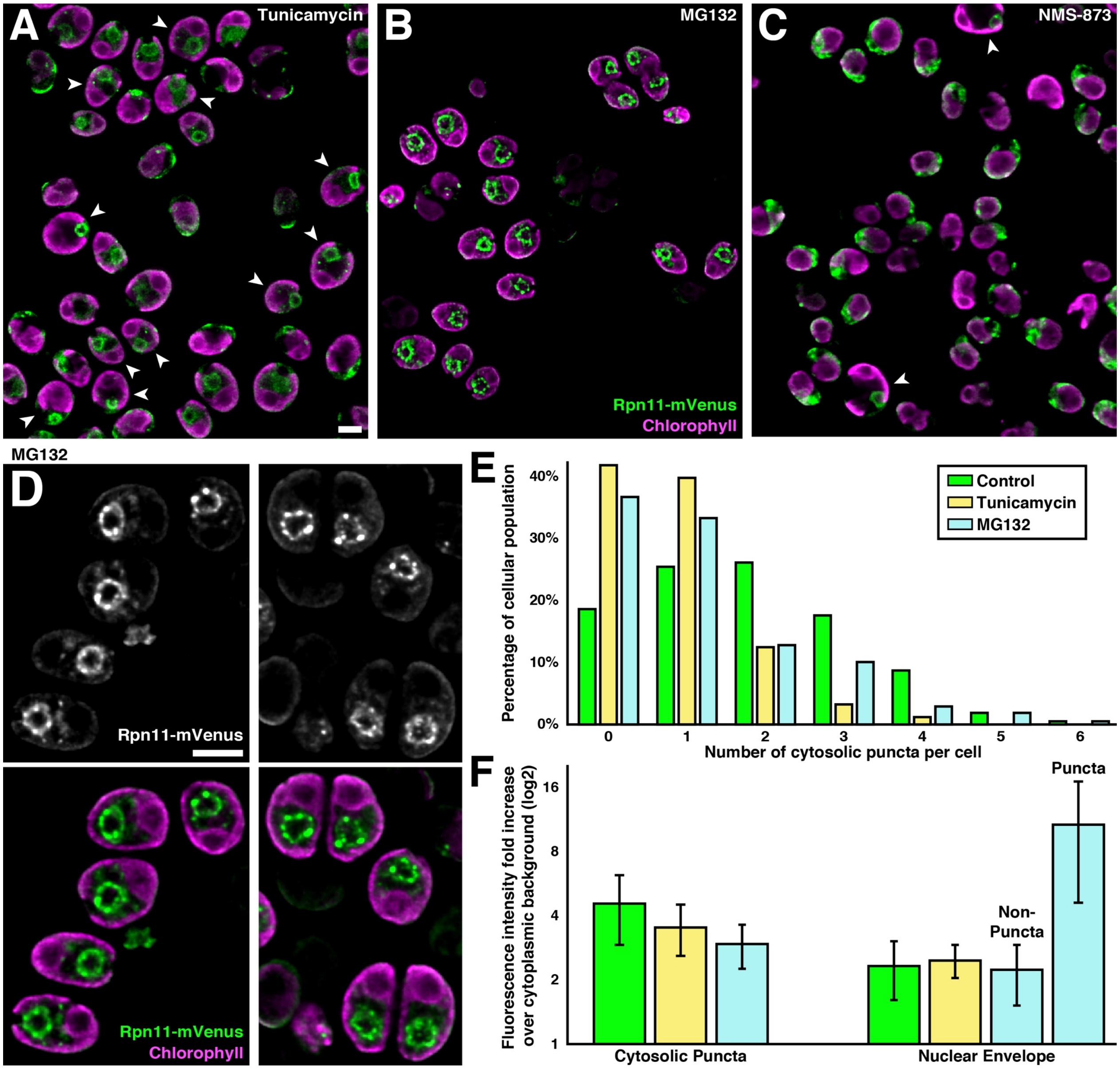


**Figure S14. Effects of pharmacological inhibitors on proteasome localization. (A-D)** Live *Chlamydomonas mat3-4* cells expressing the tagged proteasome subunit Rpn11-mVenus, imaged in 3D by widefield deconvolution fluorescence microscopy. Prior to imaging, cells were treated for 2 hr with either (**A**) 5 µg/mL tunicamycin to induce acute ER stress, (**B, D**) 50 µM MG132 to inhibit proteasome function, or (**C**) 5 µg/mL NMS-873 to inhibit Cdc48 function. Panel **D** shows enlarged views of the MG132 treatment (top: greyscale, bottom: two-color overlay) to more clearly illustrate the accumulation of bright proteasome puncta at the nuclear envelope. All images are maximum intensity projections. White arrowheads mark cells where the nucleus has been displaced from its central position by swelling of the vacuole, indicating toxic effects of tunicamycin and NMS-873. Scale bars in **A** and **D**: 5 µm. **(E)** Histogram of the number of cytosolic puncta per cell. N= 213, 149, and 192 cells for control, tunicamycin, and MG132, respectively. **(E)** The intensity of Rpn11-mVenus fluorescence within the cytosolic puncta and at the nuclear envelope, normalized by fold-change over each cell's cytosolic background. Quantification of

MG132 was separated into regions of the nuclear envelope that are not occupied by bright puncta (Non-Puncta) and regions that do contain puncta (Puncta). The y-axis is plotted in log2 scale. Error bars show standard deviation.

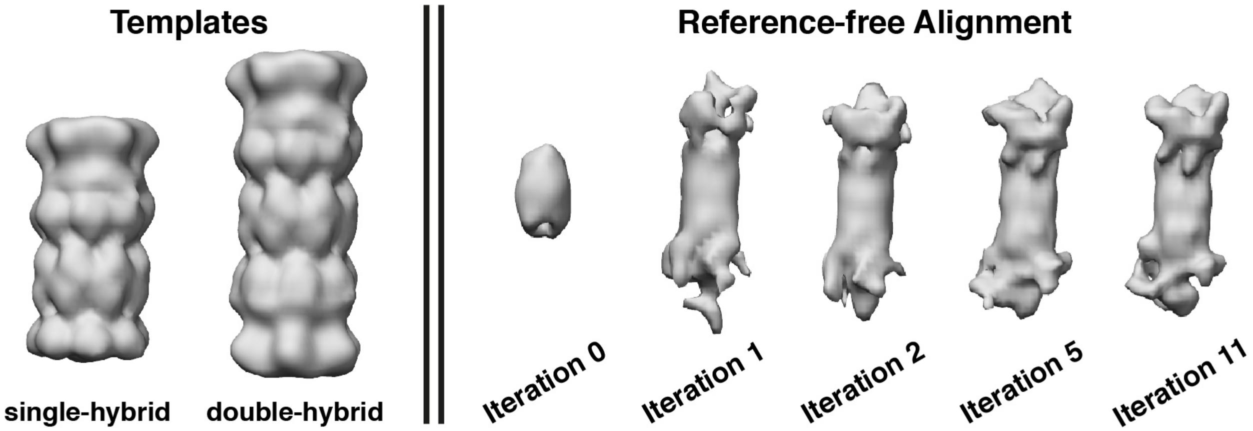


**Figure S15. Hybrid Cdc48-proteasome complexes are not found within the cellular tomograms by template matching. Left:** the tomograms where searched with templates of a 20S proteasome core particle attached on its ends to one (single-hybrid) or two (double-hybrid) Cdc48 complexes. **Right:** the particles with the top scoring correlation coefficients were extracted and subjected to reference-free alignment in PyTom software (10). Rather than recovering a hybrid structure, these particles aligned to produce a structure of a normal 26S proteasome.

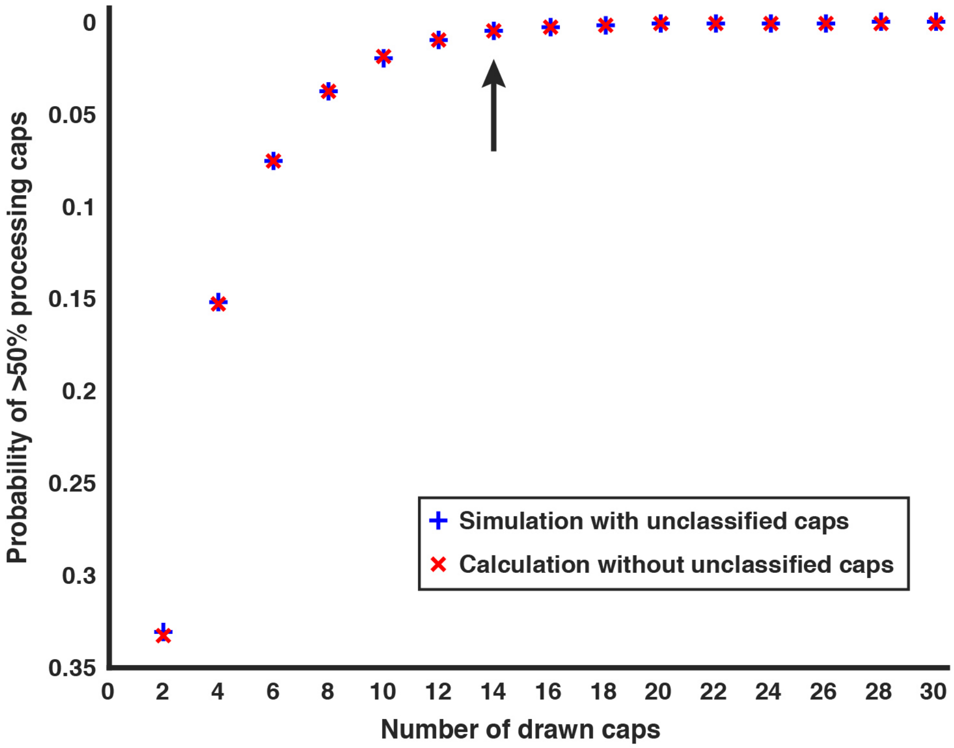


**Figure S16. Numerical justification for omitting unclassified caps from the statistical test of proteasome activity close to the ER membrane (see methods and Fig. 5B).** The probability of drawing a group of proteasomes with >50% substrate-processing caps from the pool of clustered proteasomes >20 nm from the membrane, evaluated over different sample sizes. In blue, the pool is comprised of ground, processing, and unclassified caps, and the probability was determined by numerical simulations (1000 draws). In red, the probability was analytically calculated from a pool of only ground and processing caps, using an urn model without replacement and order. The arrow indicates the number of caps <20 nm from the membrane in our dataset (sample size= 14). The numerical and analytical results overlap, suggesting that the unclassified caps can be discarded without affecting the probability.

**Table S1. Homologs to yeast, human, and *Arabidopsis thaliana* ERAD components identified in the *Chlamydomonas reinhardtii* genome** (11, 12). *Chlamydomonas* and *Arabidopsis* gene accessions numbers are from the Phytozome platform (1). Accompanies Figs. S1-S2.

| | *S. cerevisiae* | *H. sapiens* | *A. thaliana* | *C. reinhardtii* | **Description** |
|---|---|---|---|---|---|
| **E3-related** | Hrd1 / Der3 | HRD1<br>Gp78 / AMFR | At3g16090<br>At1g65040 | Cre04.g217922 | E3 ligase and ER retrotranslocation channel, primarily performs ERAD of luminal and membrane proteins (ERAD-L and ERAD-M). **See notes 1 and 2.** |
| | Hrd3 | SEL1L | At1g18260<br>At1g73570 | Cre03.g197400 | Forms heterodimer with Hrd1, involved in substrate recognition. **See note 2.** |
| | Doa10 | TEB4 /<br>MARCH-VI | At4g34100 | Cre12.g517800<br>Cre12.g517850 | E3 ligase, primarily performs ERAD of proteins with misfolded cytosolic domains (ERAD-C). **See note 3.** |
| | Usa1 | HERP | Not Found | Not Found | Oligomerization scaffold, binds Hrd1 and recruits Der1. **See notes 1 and 4.** |
| | Der1<br>Dfm1 | Derlin-1<br>Derlin-2<br>Derlin-3 | At4g29330<br>At4g04860 | Cre15.g636400<br>Cre12.g523500 | Forms complexes with Hrd1, Doa10, and Cdc48. Required for ERAD of certain substrates. |
| **E2-related** | Cue1 | CueDC1 /<br>CueD1 | At1g27752 | Cre13.g568750 | Recruits Ubc7 to the ER, targeting it to Hrd1 and Doa10. **See note 5.** |
| | Ubc7 | Ube2g1<br>Ube2g2 | At3g55380 | Cre12.g546650 | E2 enzyme, functions with Hrd1 and Doa10. |
| | Ubc6 | Ube2j1<br>Ube2j2 | At3g17000 | Cre08.g372400 | E2 enzyme, functions with Doa10. |
| **Substrate-related** | Pdi1 | ERdj5 | At1g77510<br>At3g54960<br>At5g60640 | Cre02.g088200 | Protein disulfide isomerase, involved in substrate recognition, reduces disulfide bonds on misfolded ER proteins. |
| | Htm1 / Mnl1 | EDEM1 | At5g43710<br>At1g27520 | Cre06.g301600 | α-mannosidase, involved with substrate recognition, interacts with Pdi1 and removes mannose residue to create α-1,6 mannose linkage. |
| | Yos9 | OS-9<br>XTB3-B | At5g35080 | Cre06.g257900 | Lectin, involved with substrate recognition, recognizes α-1,6 mannose linkage, targeting misfolded protein for ERAD. Interacts with Hrd3. |
| | Kar2 | BiP | At5g42020<br>At5g28540<br>At1g09080 | Cre02.g080700<br>Cre02.g080600 | ER Hsp70 chaperone, involved in substrate recognition, helps deliver misfolded proteins to Hrd1-Hrd3. **See note 6.** |
| **Cdc48-related** | Ubx2 | UBXD2<br>UBXD8 / FAF2 | At4g10790 | Cre03.g200100 | Cdc48 adaptor, recruits the Cdc48-Npl4-Udf1 complex to the ER. |
| | Npl4 | NPL4 | At3g63000 | Cre06.g293051 | Cdc48 ERAD-specific cofactor, forms heterodimer with Udf1. **See note 7.** |
| | Ufd1 | UFD1L | At2g21270<br>At4g38930 | Cre14.g613350<br>Cre03.g179100 | Cdc48 ERAD-specific cofactor, forms heterodimer with Npl4. |
| | Cdc48 | p97 / VCP | At3g53230<br>At5g03340 | Cre06.g269950 | AAA-type ATPase, extracts proteins from the ER membrane. |

**Note 1:** The predicted *Chlamydomonas* Hrd1 homolog, Cre04.g217922, is 951 aa, compared to 551 aa for yeast and 460 aa for *Arabidopsis*. However, the current gene model overlaps with a transposon-like insertion between the last two exons, likely responsible for an incorrect gene model containing a long C-terminal stretch of low-complexity sequence. GrabB seed extension (2)

corrected the gene model to 333 aa, containing six transmembrane domains and ending after the RING domain (SI Appendix, Fig. S2). The corrected gene model is missing the C-terminal domain of Hrd1 that binds Usa1. Interestingly, a homolog for Usa1 could not be found in the *Chlamydomonas* genome (see note 4).

**Note 2:** The *Arabidopsis* genome possesses two genes with significant similarity to Hrd1 and two genes with significant similarity to Hrd3. In plants, ERAD is involved in the targeted degradation of two mutant alleles of the brassinosteroid receptor BRI1; *bri1-5* and *bri1-9* missense mutants cause ER-retention and subsequent ERAD of the receptor. A genetic screen for suppressors of *bri1-9* identified At1g18260, one of the homologs for Hrd3. The second homologue of Hrd3, At1g73570, cannot suppress *bri1-9*, suggesting it is not involved in ERAD of BRI1. The two *Arabidopsis* homologs of Hrd1, At3g16090 (HRD1A) and At1g65040 (HRD1B) are redundant, and only the *hrd1a hrd1b* double mutant can suppress *bri1-9* (13).

**Note 3:** Compared to the 1319 aa yeast Doa10, which has one ring finger and 14 transmembrane domains, Cre12.g517800 is only 298 aa, with one ring finger and four transmembrane domains. The gene immediately downstream, Cre12.g517850, shares similarity (49% identity and 60% similarity overall), with one ring finger and three transmembrane domains. The strongest sequence conservation is seen over the ring finger, with 80% identity and 88% similarity. There is no evidence that these two genes are a single locus.

**Note 4:** *Chlamydomonas* appears to lack a clear homolog for Usa1/HERP. However, in yeast RAD23 has been reported to play a role in ERAD and may substitute for Usa1/HERP. The *Chlamydomonas* genome does encode one homolog for RAD23/HR23, with a very similar protein domain architecture (SI Appendix, Fig. S1).

**Note 5:** Whereas yeast have several Cue proteins, humans only have one with just the Cue domain (CueDC1 / CueD1). There are other Cue-domain containing human proteins that contain additional domains. *Arabidopsis* and *Chlamydomonas* each only have one protein with a single Cue domain. However, the primary sequences of these Cue-encoding genes are sufficiently divergent from yeast Cue1 to have been missed by BLAST searches, and it is unknown whether they are functional homologues.

**Note 6:** *Chlamydomonas* has two orthologs for BiP: BiP1 (Cre02.g080700) and BiP2 (Cre02.g080600). BiP1 and BiP2 share 90% identity (93% similarity) over the entire length of the protein sequence. The BiP1 and BiP2 genes are within 10 kbp of each other on chromosome 2, with HSP90B (Cre02.g080650) in between.

**Note 7:** The current sequence of Cre06.g293051 includes a gap that separates two exons by an intron of 6000 Ns, flanked on either side by low complexity sequences. As a result, the current gene model for Npl40 has been fused at its N-terminus with an unrelated N-terminal ammonium transporter. GRAbB seed extension (2) corrected the gene model to 426 amino acids, containing three NPL4 domains (SI Appendix, Fig. S2).

## Supplementary Movies

**Movie 1. Cytosolic proteasome puncta dynamically assemble.** Time series of live *Chlamydomonas mat3-4* cells expressing Rpn11-mVenus. Cells were images for 14 minutes. 3D Z-stacks were acquired once per minute by widefield deconvolution fluorescence microscopy. The movies show maximum intensity projections through all Z-slices containing puncta**.** Small yellow arrowheads indicate newly assembled puncta, with clear signal that appears and can be tracked over time, despite significant photobleaching during the time series. The large red arrowhead in D is a fusion event between the newly assembled punctum and a preexisting punctum. Single frames from cells A-D are displayed in Fig. S3.

**Movie 2. Cytosolic proteasome puncta dynamically fuse.** Time series of live *Chlamydomonas mat3-4* cells expressing Rpn11-mVenus. Cells were images for 14 minutes. 3D Z-stacks were acquired once per minute by widefield deconvolution fluorescence microscopy. The movies show maximum intensity projections through all Z-slices containing puncta. Small yellow arrowheads indicate two puncta before fusion. Large red arrowheads indicate puncta after fusion. Despite significant photobleaching during the time series, fused puncta often have increased intensity due to the accumulation of more fluorescent protein within a diffraction-limited spot. Single frames from cells A-D are displayed in Fig. S4.

**Movie 3. Proteasomes and Cdc48 form ribosome-excluding degradation microcompartments that contact a specialized patch on the ER membrane.** Sequential sections back and forth through the tomographic volume shown in Fig. 2A (orthographic view), followed by reveal of the 3D segmentation from Fig. 2D (perspective view). Golgi: dark grey, ER: light grey, other organelles: white, proteasomes: red, Cdc48: yellow, free cytosolic ribosomes: light blue, membrane-bound ribosomes: dark blue. A close-up view rotates around the microcompartment, then the proteasomes are removed to show the ribosome-free patch on the ER membrane, then free cytosolic ribosomes are removed to show the clustering behavior of the proteasomes.

**Movie 4. Accurate identification of individual proteasome and Cdc48 structures within the cell.** Z-slices through the tomogram region shown in Fig. 2B-C, with transparent red and yellow silhouettes corresponding to the mapped in subtomogram averages of proteasomes and Cdc48, as shown in Fig. 2D. A clipping plane has been used to only show the averages that are just above each tomogram Z-slice, allowing comparison between the placed averages and the tomogram. Accompanies Fig. S5.

**Author contributions**:

W.W. and C.L. created transgenic strains and performed the fluorescence imaging and analysis. M.S. performed the FIB milling. M.S., S.A., and B.D.E. acquired the tomograms. S.A. performed all cryo-ET image analysis in the paper, with assistance from F.B., B.D.E., and J.S. Proteomics was performed by N.N. (MPI-B core facility) and S.A., while P.A.S. and J.S. performed the bioinformatics analysis. B.D.E., J.M.P, and W.B. conceived and supervised the project. S.A., J.S.,W.B., and B.D.E. wrote the paper, with input from all authors.

**Supplementary References**


1. Goodstein DM*, et al.* (2012) Phytozome: a comparative platform for green plant genomics. *Nucleic acids research* 40(Database issue):D1178-1186.

2. Brankovics B*, et al.* (2016) GRAbB: Selective Assembly of Genomic Regions, a New Niche for Genomic Research. *PLoS computational biology* 12(6):e1004753.

3. Wehmer M*, et al.* (2017) Structural insights into the functional cycle of the ATPase module of the 26S proteasome. *Proceedings of the National Academy of Sciences of the United States of America* 114(6):1305-1310.

4. Banerjee S*, et al.* (2016) 2.3 A resolution cryo-EM structure of human p97 and mechanism of allosteric inhibition. *Science (New York, N.Y.)* 351(6275):871-875.

5. Svidritskiy E, Brilot Axel F, Koh Cha S, Grigorieff N, & Korostelev Andrei A (2014) Structures of Yeast 80S Ribosome-tRNA Complexes in the Rotated and Nonrotated Conformations. *Structure* 22(8):1210-1218.

6. Huang R*, et al.* (2016) Unfolding the mechanism of the AAA+ unfoldase VAT by a combined cryo-EM, solution NMR study. *Proceedings of the National Academy of Sciences of the United States of America* 113(29):E4190-4199.

7. Guo Q*, et al.* (2018) In Situ Structure of Neuronal C9orf72 Poly-GA Aggregates Reveals Proteasome Recruitment. *Cell* 172(4):696-705.e612.

8. Albert S*, et al.* (2017) Proteasomes tether to two distinct sites at the nuclear pore complex. *Proceedings of the National Academy of Sciences of the United States of America* 114(52):13726-13731.

9. Goddard TD, Huang CC, & Ferrin TE (2007) Visualizing density maps with UCSF Chimera. *Journal of structural biology* 157(1):281-287.

10. Hrabe T*, et al.* (2012) PyTom: a python-based toolbox for localization of macromolecules in cryo-electron tomograms and subtomogram analysis. *Journal of structural biology* 178(2):177-188.

11. Merchant SS*, et al.* (2007) The Chlamydomonas genome reveals the evolution of key animal and plant functions. *Science (New York, N.Y.)* 318(5848):245-250.

12. Blaby IK*, et al.* (2014) The Chlamydomonas genome project: a decade on. *Trends in plant science* 19(10):672-680.

13. Su W, Liu Y, Xia Y, Hong Z, & Li J (2011) Conserved endoplasmic reticulum-associated degradation system to eliminate mutated receptor-like kinases in Arabidopsis. *Proceedings of the National Academy of Sciences* 108(2):870-875.
